# Efficacy and Safety of Tranexamic Acid in Abdominoplasty: A Systematic Review and Meta-Analysis

**DOI:** 10.3390/jcm15176886

**Published:** 2026-09-05

**Authors:** Kholoud Almahzoum, Abdulaziz Alenezi, Refah Fahad Alajmi, Omar W. Alrumaih, Bader N. Almutawaa, Ebraheem Albazee, Khaled Altarrah

**Affiliations:** 1 Department of General Surgery, Sheikh Jaber Al-Ahmad Al-Sabah Hospital, Ministry of Health, Kuwait City 13018, Kuwait; 2 Department of Plastic and Reconstructive Surgery, Sheikh Jaber Al-Ahmad Al-Sabah Hospital, Kuwait City 13018, Kuwait; 3 Kuwait Institute for Medical Specializations, Kuwait City 12050, Kuwait; 4 School of Medicine, The University of Jordan, Amman 11942, Jordan; 5 Department of General Surgery, Amiri Hospital Kuwait, Ministry of Health, Kuwait City 15300, Kuwait; 6 Otorhinolaryngology-Head and Neck Surgery, Kuwait Institute for Medical Specializations (KIMS), Kuwait City 13018, Kuwait; 7 Department of Otolaryngology-Head and Neck Surgery, Al-Jahra Hospital, Al-Jahra 03200, Kuwait

**Keywords:** tranexamic acid, abdominoplasty, postoperative drainage, wound complications, seroma

## Abstract

**Background**: Abdominoplasty is associated with postoperative bleeding, drainage, and seroma. **Methods**: We searched four databases from inception through 16 May 2026 for randomized and non-randomized comparative studies of systemic or topical tranexamic acid (TXA). **Results**: Seven studies (one randomized controlled trial and six observational studies) reported 763 participants across source cohorts; treatment-arm assignment was available for 753. Random-effects meta-analysis associated TXA with lower total drainage volume (MD: −233.32 mL, 95% CI: −351.43 to −115.21), shorter drainage time (MD: −1.36 days, 95% CI: −1.75 to −0.97), shorter hospital stay (MD: −1.48 days, 95% CI: −2.16 to −0.80), smaller absolute hemoglobin drop (MD: −0.87 g/dL, 95% CI: −1.72 to −0.02), and lower seroma odds (OR: 0.51, 95% CI: 0.27 to 0.96). No significant differences were observed for 24 h drainage, relative hemoglobin drop, hematoma, surgical-site infection, wound-healing disorder, or transfusion. Rare adverse events were insufficiently reported. The absolute-hemoglobin and seroma associations were not robust to leave-one-out analysis, and major Doi-plot asymmetry affected several outcomes. **Conclusions**: TXA may improve selected early outcomes, but the small, mainly observational evidence base supports cautious individualized use rather than a universal regimen.

## 1. Introduction

Abdominoplasty is a common abdominal contouring procedure that removes excess abdominal skin and fat and may include tightening of the abdominal wall [1]. It is performed for aesthetic, reconstructive, and functional indications, especially after pregnancy, massive weight loss, or abdominal wall laxity [2]. Abdominoplasty includes several operative variants, including classical, post-bariatric, and fleur-de-lis abdominoplasty, all of which share wide soft-tissue dissection, dead-space formation, and wound-healing concerns [2]. Despite their clinical value, these procedures are associated with relevant postoperative morbidity because of wide tissue dissection, dead-space formation, and large wound surfaces [3]. Seroma, hematoma, infection, wound dehiscence, skin necrosis, and delayed healing are among the most important complications after abdominoplasty [3]. Seroma is particularly important because it may cause pain, anxiety, infection, wound breakdown, repeated aspirations and delayed recovery [4]. Postoperative drainage volume and drain duration are also clinically relevant because they affect mobility, comfort, follow-up burden, and length of hospital stay [5]. Therefore, safe strategies that reduce bleeding, drainage, and wound-related complications may improve outcomes after abdominoplasty [6].

Tranexamic acid (TXA) is an antifibrinolytic agent derived from lysine that limits clot breakdown by interfering with plasminogen activation, thereby helping to preserve fibrin stability and reduce blood loss [7]. It has been widely studied in surgery and strong evidence shows that it reduces blood transfusion requirements in many operative settings [8]. Updated meta-analyses in bariatric surgery and endoscopic sinus surgery have also reported reductions in bleeding-related outcomes without a clear thromboembolic safety signal, supporting the broader blood-sparing rationale for TXA while highlighting procedure-specific evidence needs [9,10]. In aesthetic plastic surgery, TXA has gained attention as a simple and low-cost method to reduce blood loss, bruising, drainage output, and hematoma formation [11]. Recent body-contouring evidence suggests that TXA may reduce hematoma, wound-healing problems and infection risk, with no clear increase in reported thromboembolic complications [6]. However, the evidence in abdominoplasty remains inconsistent, as some studies report reduced drainage volume, complications, and hospital stay, whereas others show no significant benefit [5,12]. These differences may be related to variations in dose, route, timing, surgical technique, patient selection, and outcome definitions. Drainage and seroma may also be influenced by drain-removal criteria, quilting or progressive-tension sutures, Scarpa fascia preservation, compression, liposuction, tumescent infiltration, and electrocautery; these co-interventions can confound comparisons of TXA. Previous reviews often combined abdominoplasty with broader aesthetic-, breast-, or body-contouring procedures, limiting procedure-specific conclusions. We therefore systematically reviewed and pooled the available evidence on the efficacy and safety of TXA in abdominoplasty and examined clinically relevant regimens and surgical co-intervention heterogeneity.

## 2. Methods

### 2.1. Study Design

This systematic review synthesized evidence from comparative studies evaluating the clinical value and safety of TXA in abdominoplasty. It compared patients treated with TXA with patients receiving a placebo, no TXA, or standard perioperative care. The review followed the PRISMA 2020 reporting framework (see the PRISMA Checklist in the Supplementary Materials) and Cochrane guidance [13,14]. The protocol was prospectively registered in PROSPERO (CRD420261407362).

### 2.2. Data Sources and Search Strategy

A systematic search was conducted in Scopus, Web of Science, PubMed, and the Cochrane Library from database inception to 16 May 2026. The search was not restricted by language, geographic setting, or publication year. The search strategy combined terms for abdominoplasty with terms for TXA. The surgical terms included “abdominoplasty,” “abdominoplasties,” and the MeSH term “Abdominoplasty.” The intervention terms included “tranexamic acid,” the MeSH term “Tranexamic Acid,” and common drug names such as “Transamin,” “Amchafibrin,” “Ugurol,” “Cyklokapron,” “Anvitoff,” “Spotof,” and “Exacyl.” Full database-specific search strings are presented in Supplementary Table S1.

### 2.3. Eligibility Criteria

We considered studies suitable when they enrolled patients undergoing abdominoplasty surgery and compared TXA with a control condition. Eligible procedures included classical abdominoplasty, post-bariatric abdominoplasty, and fleur-de-lis abdominoplasty. Both intravenous and topical TXA regimens were accepted, regardless of dose, frequency, or timing. Control groups could receive a placebo, no TXA or routine care without TXA. Studies had to report at least one outcome of clinical value, including postoperative drainage, drain duration, hospital stay, hemoglobin or hematocrit change, seroma, hematoma, wound-healing disorder, surgical-site infection (SSI), transfusion, thromboembolic events, or other adverse events. The review accepted randomized studies as well as comparative non-randomized studies. Reviews, protocols, case reports, non-comparative studies, duplicate reports, studies of unrelated procedures, and reports without extractable comparative data were excluded.

### 2.4. Screening and Data Extraction

All documents exported from the database search were transferred to EndNote for duplicate screening [15]. Titles and abstracts were then reviewed, and articles that appeared potentially relevant were assessed in full text using Rayyan web platform (Rayyan Systems Inc., Cambridge, MA, USA; continuously updated; no fixed version; accessed 16 May 2026) [16]. Any differences between reviewers were settled through reviewer discussion, and final decisions were agreed before data extraction.

Information was collected using a predefined extraction form, including study name, year, country, design, study period, sample size, surgical procedure, TXA route, dose, timing, comparator, baseline characteristics, reported outcomes, and seroma-related co-interventions. The latter included drain type, number, and removal threshold; quilting or progressive-tension sutures; Scarpa fascia preservation; compression; liposuction; tumescent infiltration; and electrocautery when reported. For continuous endpoints, means, standard deviations (SDs), and total sample sizes were recorded whenever available. For binary variables, event counts and group totals were recorded. If a study reported an outcome incompletely or in a format unsuitable for pooling, it was described narratively when appropriate but excluded from that meta-analysis.

### 2.5. Outcomes

The main efficacy outcomes were drainage-related endpoints, including 24 h drainage volume, total drainage volume, and drainage time. Other pooled efficacy and recovery outcomes included hospital stay, absolute hemoglobin drop, and relative hemoglobin drop. Pooled complication outcomes included seroma, hematoma, SSI, wound-healing disorder, and blood transfusion. Safety outcomes, including venous thromboembolism, seizures, allergic reactions, and other reported TXA-related adverse events, were summarized narratively because they were uncommon and not reported consistently enough for a meta-analysis.

### 2.6. Risk-of-Bias Assessment

The RCT was assessed using the Cochrane Risk of Bias 2 (Rob-2) tool [17]. This assessment considered bias related to randomization, deviations from assigned intervention, missing data, outcome measurement, and selective reporting. We used the Newcastle–Ottawa Scale (NOS) to judge the methodological quality of observational studies across three main domains: cohort selection, comparability, and outcome assessment [18].

### 2.7. Statistical Analysis

Statistical analyses were conducted in R version 4.6.1 (R Foundation for Statistical Computing, Vienna, Austria) using the meta (version 8.5-0) and dmetar (version 0.0.9000) packages [19,20]. Continuous outcomes were pooled as MDs with 95% confidence intervals (CIs), and binary outcomes were expressed as odds ratios (ORs) with 95% CIs. Random-effects models were used because variation in surgical technique, TXA regimen, route, patient population, comparator, and outcome definition was expected. Statistical heterogeneity was evaluated using I^2^ and τ^2^. Leave-one-out sensitivity analyses were conducted when feasible. In response to study-quality concerns, a post hoc quality-based sensitivity analysis excluded any study rated as poor quality or at a high risk of bias from each applicable meta-analysis. Doi plots and LFK indices were used exploratorily to assess small-study effects; because most analyses included very few studies, these findings were treated as signals rather than diagnostic evidence [21].

## 3. Results

### 3.1. Selection Process

**Figure 1:**
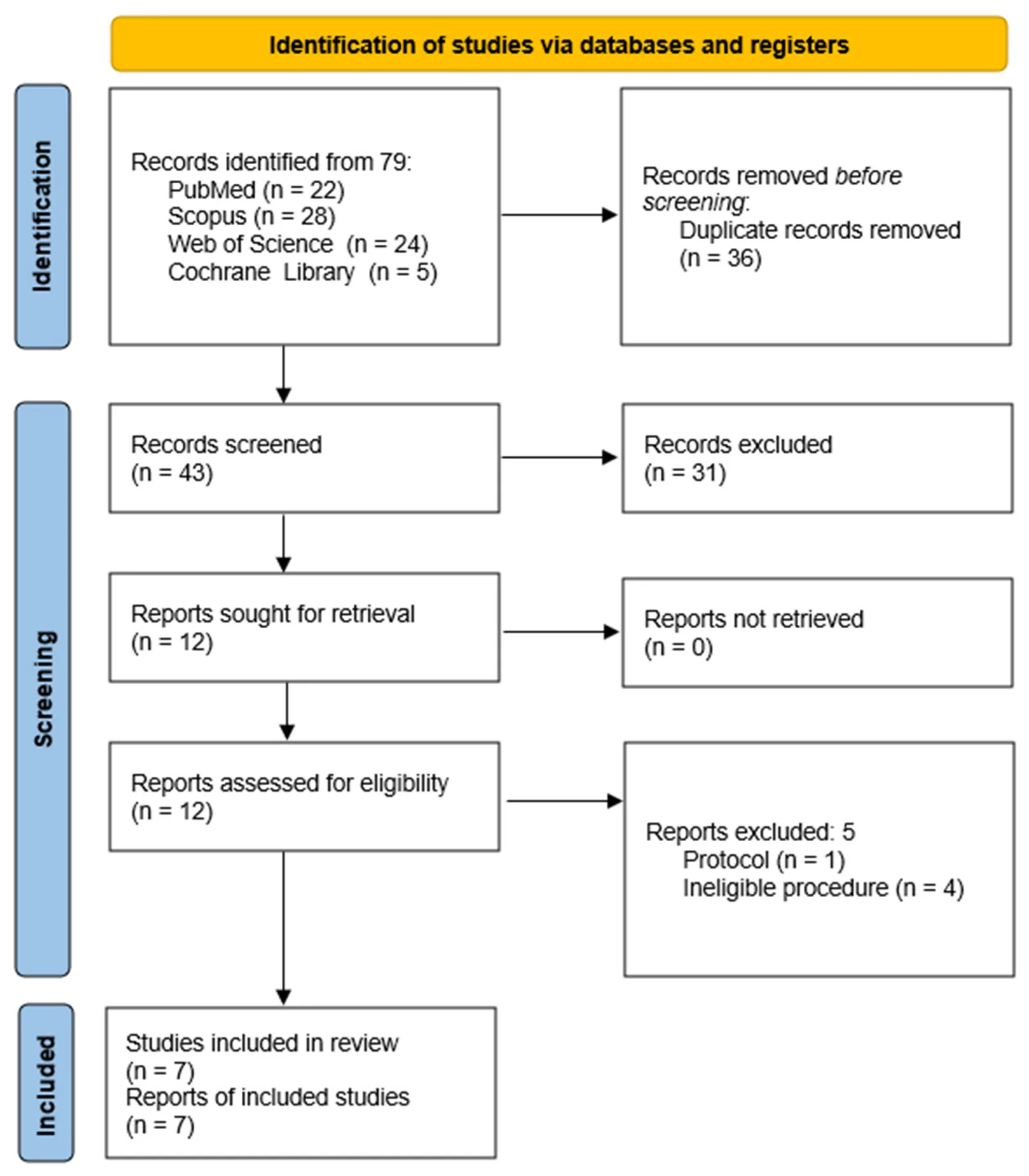
PRISMA flow diagram of the study selection process.

Database searching retrieved 79 citations: 22 from PubMed, 28 from Scopus, 24 from Web of Science, and 5 from the Cochrane Library. After duplicate removal, 43 records underwent title and abstract screening, and 31 were excluded. The remaining 12 reports were reviewed in full text, all of which were available. Five reports were excluded: one protocol study and four studies with ineligible procedures. Seven studies were included [5,12,22,23,24,25,26]. The selection process is shown in Figure 1.

### 3.2. Characteristics of the Included Studies

Seven studies originating from Germany, Austria, Poland, Italy, and Switzerland were included. One was a randomized controlled trial and six were comparative observational studies. The source cohorts reported 763 participants in total, and treatment-arm assignment was available for 753. Specifically, Manfellotto 2025 [24] reported 221 participants overall but only 181 TXA-treated and 30 untreated participants by arm; the remaining 10 were not classified by TXA status in the source report. No allocation was imputed, and outcome-specific denominators were used. The procedures included elective, classical, and post-bariatric abdominoplasty. Control groups received a placebo, no TXA, or routine care without TXA (Table 1 and Table 2).

TXA dosing was not uniform across studies. Zaussinger 2024 [25] used bodyweight-adapted 1–2 g IV after resection and before closure; Kolasiński 2026 [12] used a single 10 mg/kg IV dose completed at least 10 min before incision; Manfellotto 2025 [24] used 1 g IV preoperatively; Sahinovic 2025 [5] used 1 g IV at incision followed by a source-reported “1-1-1” regimen for 48 h, with oral continuation after early discharge; Zucal 2025 [26] used 1 g of topical TXA in 20 mL of saline through the drains, which were clamped for 20 min; Ciccarelli 2026 [22] used 1 g IV at induction plus 500 mg local infiltration, followed by 2 g IV on postoperative day 1 and 1 g IV on postoperative day 2; and Gorji 2026 [23] used 1 g IV at the start of surgery. Thus, no common dose, route, timing, or frequency was used [5,12,22,23,24,25,26]. The full regimen details are presented in Table 1 and Supplementary Table S3.

Seroma-related co-interventions were also clinically heterogeneous and incompletely reported. Drain-removal thresholds varied from 20 to 30 mL/24 h when specified, whereas Gorji 2026 [23] used output and clinical judgment without a strict threshold. Quilting or progressive-tension sutures, Scarpa fascia preservation, compression, liposuction, tumescent infiltration, and electrocautery practices differed or were unreported across studies (Supplementary Table S3).

### 3.3. Quality Assessment

Kolasiński 2026 was assessed using the Rob-2 tool and was judged to have a low risk of bias across all domains [12]. Among the six observational studies assessed by the NOS, five were rated as good quality [5,22,23,25,26]. Manfellotto 2025 was rated as poor quality because of limitations in selection and comparability [24]. The risk-of-bias assessment for the RCT is shown in Supplementary Figure S1, and the NOS assessment is presented in Supplementary Table S2.

### 3.4. Outcomes

#### 3.4.1. Drainage-Related Outcomes

**Figure 2:**
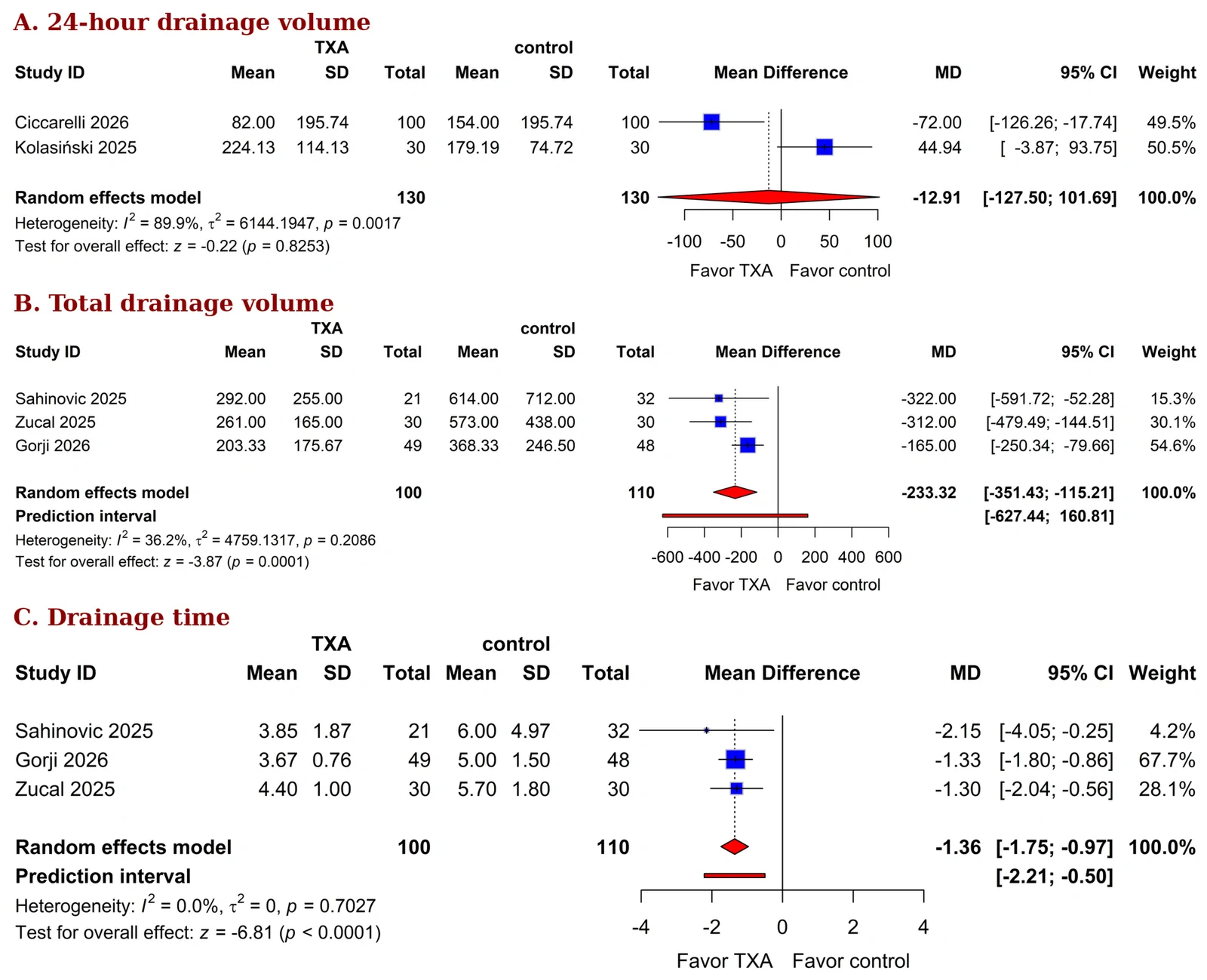
Forest plots of drainage-related outcomes: (**A**) 24 h drainage volume; (**B**) total drainage volume; (**C**) drainage time. Study labels refer to the included studies [5,12,22,23,24,25,26].

Two studies reported 24 h drainage volume. TXA was associated with a small numerical reduction, but the pooled effect was not statistically significant (MD = −12.91 mL, 95% CI: −127.50 to 101.69; *p* = 0.8253), with high heterogeneity (I^2^ = 89.9%) (Figure 2A).

Three studies reported total drainage volume. TXA was associated with significantly lower total drainage volume than the control (MD = −233.32 mL, 95% CI: −351.43 to −115.21; *p* = 0.0001), with low-to-moderate heterogeneity (I^2^ = 36.2%) (Figure 2B). This finding remained significant in the leave-one-out analysis, ranging from MD = −314.78 mL (95% CI: −457.07 to −172.50; I^2^ = 0%) after omitting Gorji 2026 to MD = −189.22 mL (95% CI: −300.36 to −78.07; I^2^ = 13%) after omitting Zucal 2025 (Supplementary Figure S2). The Doi plot showed major asymmetry (LFK index = −5.45) (Supplementary Figure S3).

Three studies reported drainage time. TXA was associated with significantly shorter drainage time than the control (MD = −1.36 days, 95% CI: −1.75 to −0.97; *p* < 0.0001), with no observed heterogeneity (I^2^ = 0.0%) (Figure 2C). The result remained robust in the leave-one-out analysis, ranging from MD = −1.41 days (95% CI: −2.10 to −0.72; I^2^ = 0%) after omitting Gorji 2026 to MD = −1.32 days (95% CI: −1.72 to −0.92; I^2^ = 0%) after omitting Sahinovic 2025 (Supplementary Figure S4). The Doi plot showed major asymmetry (LFK index = −3.46) (Supplementary Figure S5).

#### 3.4.2. Hematologic Outcomes

**Figure 3:**
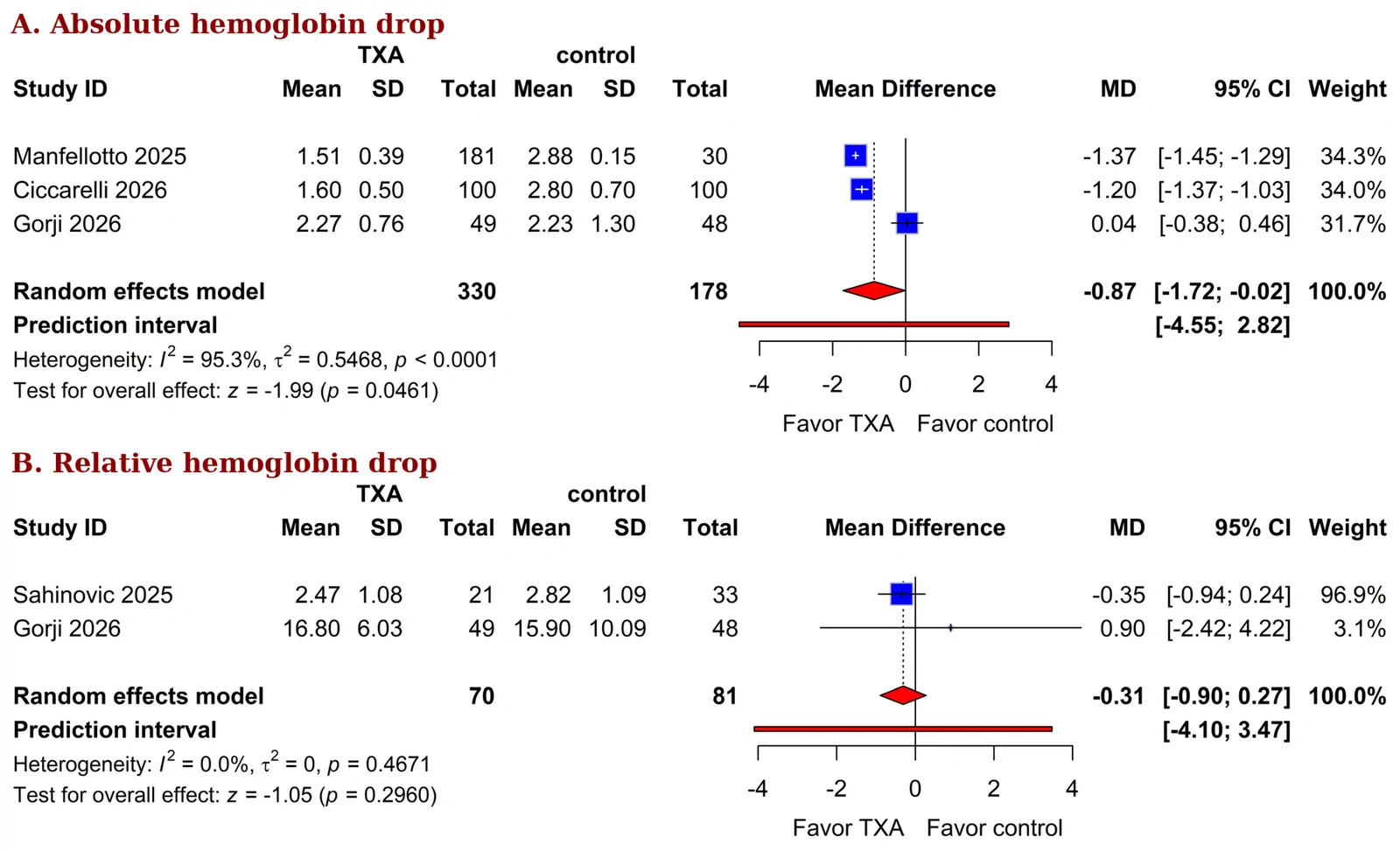
Forest plots of hematologic outcomes: (**A**) absolute hemoglobin drop; (**B**) relative hemoglobin drop. Study labels refer to the included studies [5,12,22,23,24,25,26].

Three studies reported absolute hemoglobin drop. TXA was associated with a significantly smaller decline than the control (MD = −0.87 g/dL, 95% CI: −1.72 to −0.02; *p* = 0.0461), with substantial heterogeneity (I^2^ = 95.3%) (Figure 3A). The result was not stable in the leave-one-out analysis: it remained significant after omitting Gorji 2026 (MD = −1.30 g/dL, 95% CI: −1.47 to −1.14; I^2^ = 69%) but became non-significant after omitting Manfellotto 2025 (MD = −0.60 g/dL, 95% CI: −1.81 to 0.62; I^2^ = 98%) or Ciccarelli 2026 (MD = −0.68 g/dL, 95% CI: −2.06 to 0.70; I^2^ = 98%) (Supplementary Figure S6). Because Manfellotto 2025 was the only poor-quality study and contributed only to this meta-analysis, its omission constituted the quality-based sensitivity analysis (Supplementary Table S4). No other pooled outcome included a poor-quality study. The Doi plot showed major asymmetry (LFK index = 4.94) (Supplementary Figure S7).

Two studies reported relative hemoglobin drop. The pooled analysis showed no significant difference between TXA and the control (MD = −0.31, 95% CI: −0.90 to 0.27; *p* = 0.2960), with no observed heterogeneity (I^2^ = 0.0%) (Figure 3B).

#### 3.4.3. Recovery and Fluid-Collection Outcomes

**Figure 4:**
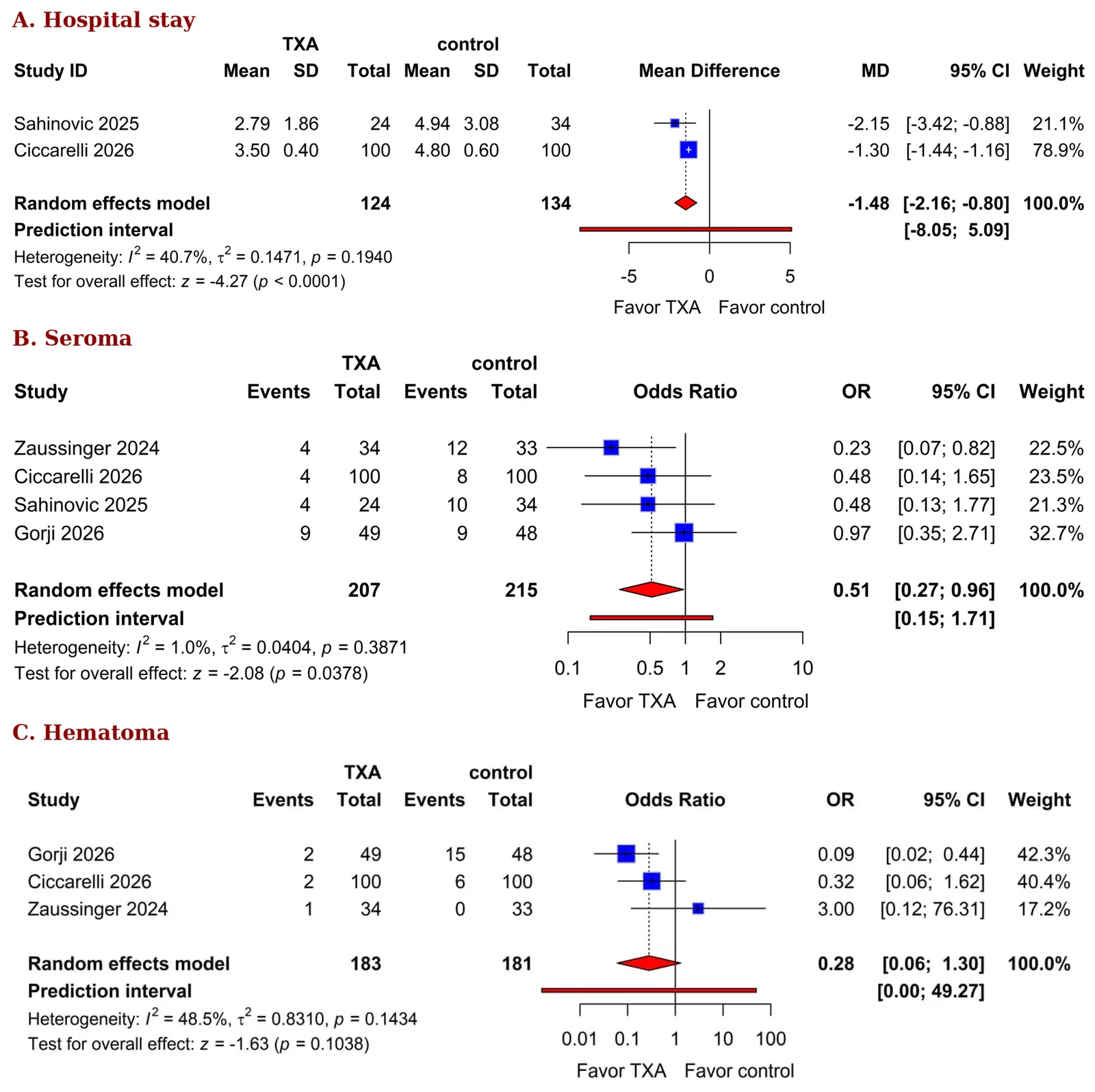
Forest plots of recovery and fluid-collection outcomes: (**A**) hospital stay; (**B**) seroma; (**C**) hematoma. Study labels refer to the included studies [5,12,22,23,24,25,26].

Two studies reported the length of hospital stay. TXA was associated with a significantly shorter stay than the control (MD = −1.48 days, 95% CI: −2.16 to −0.80; *p* < 0.0001), with moderate heterogeneity (I^2^ = 40.7%) (Figure 4A).

Four studies reported seroma. TXA was associated with lower seroma odds than the control (OR = 0.51, 95% CI: 0.27 to 0.96; *p* = 0.0378), with minimal heterogeneity (I^2^ = 1.0%) (Figure 4B). However, this result was not robust. Significance was maintained after omitting Gorji 2026 [23] (OR = 0.38, 95% CI: 0.18 to 0.78; I^2^ = 0%) but was lost after omitting Zaussinger 2024 [25] (OR = 0.65, 95% CI: 0.33 to 1.28; I^2^ = 0%), Ciccarelli 2026 [22] (OR = 0.51, 95% CI: 0.22 to 1.20; I^2^ = 34%), or Sahinovic 2025 [5] (OR = 0.51, 95% CI: 0.22 to 1.17; I^2^ = 34%) (Supplementary Figure S8). The Doi plot showed no important asymmetry (LFK index = −0.36) (Supplementary Figure S9).

Three studies reported hematoma. Although the pooled estimate favored TXA, the difference was not significant (OR = 0.28, 95% CI: 0.06 to 1.30; *p* = 0.1038), with moderate heterogeneity (I^2^ = 48.5%) (Figure 4C). The estimate became significant only after omitting Zaussinger 2024 [25] (OR = 0.17, 95% CI: 0.05 to 0.56; I^2^ = 13%), indicating leave-one-out instability (Supplementary Figure S10). The Doi plot showed minor asymmetry (LFK index = 1.70) (Supplementary Figure S11).

#### 3.4.4. Complication-Related Outcomes

**Figure 5:**
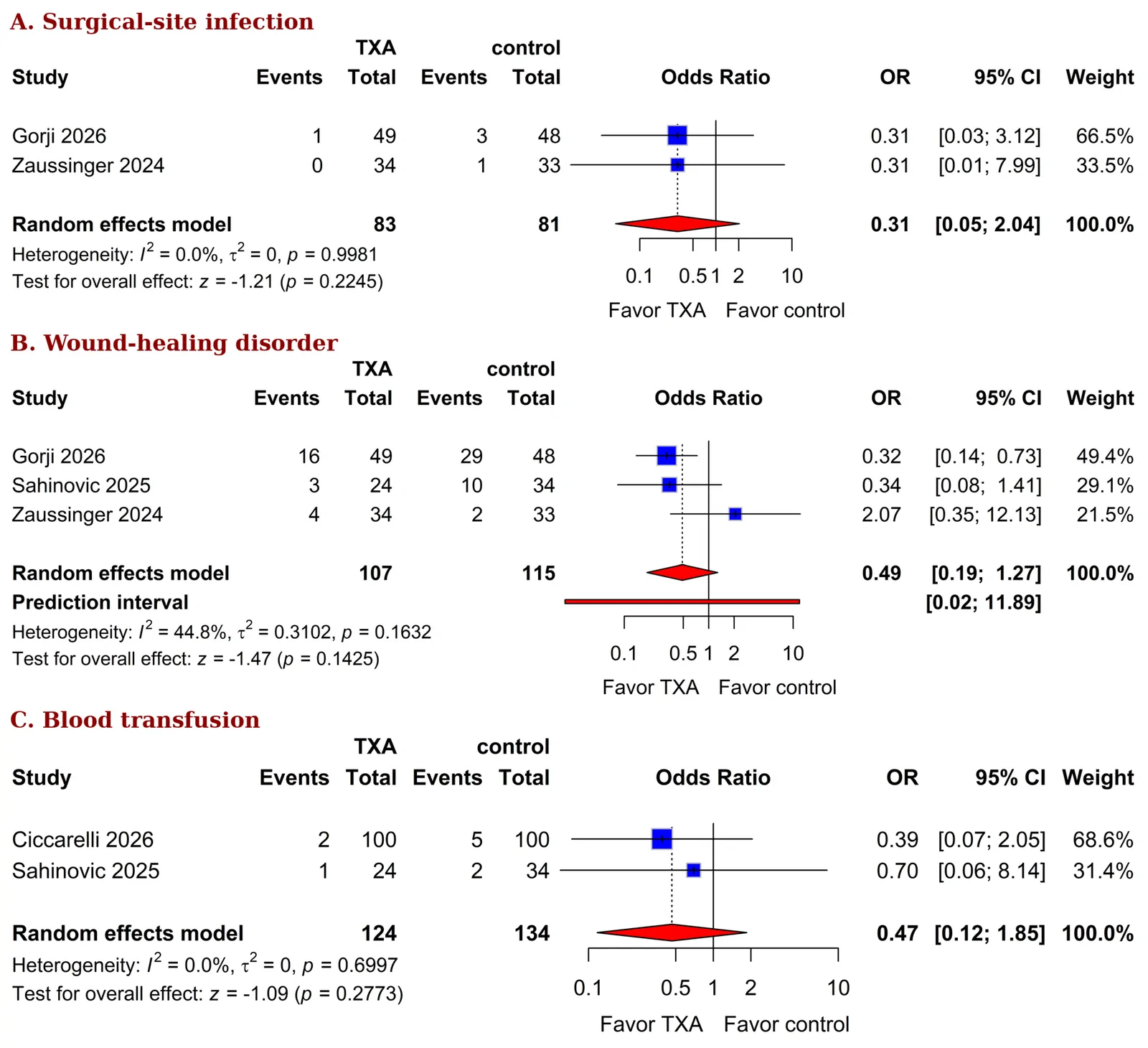
Forest plots of complication-related outcomes: (**A**) surgical-site infection; (**B**) wound-healing disorder; (**C**) blood transfusion. Study labels refer to the included studies [5,12,22,23,24,25,26].

Two studies reported surgical-site infection. The pooled analysis showed no significant difference between TXA and the control (OR = 0.31, 95% CI: 0.05 to 2.04; *p* = 0.2245), with no observed heterogeneity (I^2^ = 0.0%) (Figure 5A).

Three studies reported wound-healing disorder. The pooled estimate favoured TXA but was not significant (OR = 0.49, 95% CI: 0.19 to 1.27; *p* = 0.1425), with moderate heterogeneity (I^2^ = 44.8%) (Figure 5B). The estimate became significant only after omitting Zaussinger 2024 [25] (OR = 0.32, 95% CI: 0.16 to 0.66; I^2^ = 0%), indicating sensitivity to individual-study influence (Supplementary Figure S12). The Doi plot showed major asymmetry (LFK index = 3.07) (Supplementary Figure S13).

Two studies reported blood transfusion. The pooled estimate favored TXA but was not significant (OR = 0.47, 95% CI: 0.12 to 1.85; *p* = 0.2773), with no observed heterogeneity (I^2^ = 0.0%) (Figure 5C).

#### 3.4.5. Safety Outcomes

Rare safety outcomes, including venous thromboembolism, seizures, allergic reactions, and other TXA-related adverse events, were not reported consistently enough for quantitative pooling. Across the included studies, no consistent increase in major TXA-related adverse events was observed. However, because these events were uncommon and inconsistently reported, the available evidence is insufficient to exclude rare harms.

## 4. Discussion

This review found associations between TXA and lower total drainage volume, shorter drainage time, and shorter hospital stay after abdominoplasty. Absolute hemoglobin drop and seroma were also significant in the primary models, but neither association was robust to leave-one-out analysis and both should be interpreted cautiously. TXA was not significantly associated with 24 h drainage volume, relative hemoglobin drop, hematoma, surgical-site infection, wound-healing disorder, or blood transfusion. No consistent safety signal was identified, although rare adverse events were reported too inconsistently to exclude uncommon harms.

The reduction in total drainage volume is clinically important because postoperative drains affect comfort, mobility, outpatient care, and patient anxiety [27]. This finding is biologically plausible because TXA inhibits fibrinolysis and stabilizes clot formation, which may reduce slow postoperative oozing from the large dissected surface created during abdominoplasty [28]. This result is consistent with earlier meta-analytic evidence in aesthetic plastic surgery, which showed that both systemic and topical TXA were associated with reduced total blood loss while topical TXA was also linked to lower drainage output [11]. It also agrees with the broader body-contouring meta-analysis, which reported that TXA may reduce bleeding-related complications in breast- and body-contouring procedures [6]. The non-significant effect on 24 h drainage may be justified by the composition of early drain fluid, which can include irrigation fluid, tumescent solutions, lymphatic fluid, and inflammatory exudates in addition to blood. Therefore, TXA may influence cumulative drainage more clearly than the first-day drainage value.

TXA shortened drainage time by 1.36 days. This is a practical finding because earlier drain removal can reduce patient burden and may simplify postoperative follow-up. However, drain duration is not determined by biology alone. It also depends on local removal thresholds, surgeon preference, outpatient access, and whether the procedure is performed as inpatient or outpatient surgery [29]. For this reason, the finding should be interpreted as supportive but not definitive. Future trials should use predefined drain removal criteria so that the effect of TXA on drain duration can be interpreted more reliably.

Hospital stay was also shorter with TXA. This may be secondary to reduced drainage, earlier drain removal, and improved early postoperative recovery. TXA can reduce transfusion requirements in surgical patients and may reduce hospital stay and healthcare cost [30]. However, in abdominoplasty, discharge is influenced by many factors, including pain control, mobility, drain policy, comorbidities, and social support. Therefore, our result suggests a possible recovery benefit, but it should not be interpreted as proof that TXA alone shortens admission.

The primary model suggested a smaller absolute hemoglobin drop with TXA, but heterogeneity was very high and the association was not robust. In the quality-based sensitivity analysis excluding Manfellotto 2025 [24], the only poor-quality study, the estimate became non-significant (MD = −0.60 g/dL, 95% CI: −1.81 to 0.62; I^2^ = 98%). This is compatible with a hemostatic effect but does not establish a reliable magnitude in abdominoplasty. Differences in dose, route, timing, liposuction, operative extent, baseline hemoglobin, fluid administration, and timing of postoperative testing may explain part of the heterogeneity. The non-significant relative hemoglobin result was based on fewer studies and may also be affected by perioperative fluid shifts.

Seroma odds were lower with TXA in the primary model, but the association lost significance after omission of three of the four contributing studies and therefore was not robust. Seroma reflects dead space, lymphatic disruption, flap movement, tissue trauma, electrocautery, and drain management, not bleeding alone [4]. TXA may limit postoperative oozing and fluid accumulation, but it cannot substitute for dead-space control, appropriate drain strategy, and careful technique. Published experience on seroma prevention and management likewise supports combining operative and postoperative measures rather than relying on a single adjunct [31,32].

The included studies were not clinically homogeneous with respect to seroma prevention. Quilting or progressive-tension sutures were explicitly absent in Zaussinger 2024 [25], Manfellotto 2025 [24], and Zucal 2025 [26]; variably used in Sahinovic 2025 [5]; and unreported elsewhere. Scarpa fascia preservation was described in Manfellotto 2025 [24] and partly in Zaussinger 2024 [25]. Compression, liposuction, tumescent infiltration, electrocautery, and drain-removal thresholds also varied. These co-interventions may confound drainage and seroma associations; therefore, low statistical heterogeneity should not be interpreted as evidence of operative homogeneity.

The TXA group had fewer hematoma events, but the between-group difference was not statistically significant. This direction is consistent with the antifibrinolytic mechanism of TXA and with previous body-contouring evidence, where TXA significantly reduced hematoma risk across breast- and abdominal body-contouring procedures [6]. Our non-significant result may be explained by the low number of hematoma events and the narrower focus on abdominoplasty-related procedures. Clinically, even a modest reduction in hematoma could be relevant because a hematoma may require evacuation, delay healing, and increase pain. However, the current abdominal evidence is not strong enough to claim a definite protective effect.

SSI and wound-healing disorder were not significantly reduced with TXA in the pooled analysis. Although the point estimates favored TXA, the precision of these estimates was restricted by the small number of contributing studies and wide CIs. The wound-healing disorder outcome was also sensitive to individual-study influence in leave-one-out analysis. Therefore, the available evidence is insufficient to confirm a protective effect of TXA on infection or wound-healing complications. Any possible benefit may be indirect, such as through reduced bleeding, drainage, and fluid accumulation, but confounding by surgical technique, smoking, diabetes, body mass index, antibiotic protocols, and postoperative care cannot be excluded.

Blood transfusion was not significantly reduced. This does not contradict the established blood-saving effect of TXA in major surgery. Abdominoplasty is usually an elective procedure with relatively low transfusion rates, especially when careful hemostasis and patient selection are used [33]. Therefore, transfusion may be too rare to detect a clear difference in this setting. Drainage volume, drain duration, and hemoglobin drop may be more sensitive endpoints for evaluating TXA in abdominoplasty.

Safety is important because abdominoplasty and post-bariatric body-contouring patients may already have risk factors for venous thromboembolism. In our review, no clear increase in thromboembolic or neurologic events was observed. This agrees with broader surgical evidence and professional statements suggesting that TXA reduces bleeding without a clear increase in thromboembolic events when used appropriately [34,35]. Still, the current abdominoplasty evidence is too small to exclude rare harms. TXA should therefore be used with individualized risk assessment, attention to renal function, avoidance of excessive dosing, and standard venous thromboembolism prophylaxis when indicated.

### 4.1. Clinical Implications

Current general guidance supports TXA for adults undergoing surgery in an operating theatre where bleeding is possible and skin or mucosa is breached. NICE also advises clinicians to base TXA decisions on the full clinical picture [36]. Our findings provide cautious procedure-specific support for considering TXA as an adjunct in selected abdominoplasty patients, particularly for cumulative drainage and drain duration. However, TXA should not replace established seroma-prevention measures, and the evidence does not establish a definitive effect on hematoma or transfusion. In practice, TXA should be integrated with meticulous hemostasis, dead-space control, appropriate drain strategy, compression, mobilization, and venous-thromboembolism prevention. The current evidence does not define an optimal dose or route; routine universal protocols require stronger randomized evidence.

### 4.2. Strengths, Limitations, and Recommendations

This review has several strengths. It focused specifically on abdominoplasty surgeries rather than combining many unrelated aesthetic operations. It assessed both efficacy and safety and included clinically relevant outcomes such as drainage, drain duration, hospital stay, wound problems and transfusion. It also used sensitivity and small-study assessments to identify outcomes that were more stable.

The main limitation is that only one study was randomized and most evidence was observational, increasing the risk of selection bias and confounding. No common TXA dose, route, timing, or frequency was used, so dose–response or route-specific conclusions could not be drawn. Surgical co-interventions relevant to drainage and seroma were heterogeneous and often incompletely reported, including drain thresholds, quilting or progressive-tension sutures, Scarpa fascia preservation, compression, liposuction, tumescent infiltration, and electrocautery. Outcome definitions were also inconsistent. Major Doi-plot asymmetry for total drainage volume (LFK −5.45), drainage time (−3.46), absolute hemoglobin drop (4.94), and wound-healing disorder (3.07) raises substantial concern for small-study effects, publication bias, and possible effect-size inflation. However, the very small number of studies makes LFK estimates unstable; these signals are non-diagnostic and should not be interpreted as proof of publication bias. Future multicenter randomized trials should standardize TXA regimens, operative co-interventions, drain-removal thresholds, and outcome definitions, and they should report venous-thromboembolism prophylaxis, Caprini scores, renal function, seizures, allergic reactions, reoperation, patient satisfaction, cost, and return to activity.

## 5. Conclusions

TXA was associated with lower total drainage volume, shorter drainage time, and shorter hospital stay after abdominoplasty. The associations with absolute hemoglobin drop and seroma were not robust to leave-one-out analysis and should be interpreted cautiously. TXA was not significantly associated with 24 h drainage volume, relative hemoglobin drop, hematoma, surgical-site infection, wound-healing disorder, or blood transfusion. No clear safety signal was detected, but rare adverse events were insufficiently reported. Major Doi-plot asymmetry and the small, mainly observational evidence base further limit confidence. TXA may be considered as an individualized adjunct, but the evidence does not support a definitive universal regimen.

## Figures and Tables

**Table 1:** Summary of included-study design and TXA regimens.

Study ID	Study Design	Country	Study Period	Inclusion Criteria	Total N	Intervention Group	Control Group
Zaussinger 2024 [25]	Retrospective cohort	Austria	January 2015 and April 2023	patients aged ≥18 years old who underwent abdominoplasty	67	TXA 1–2 g IV (weight-adapted) after resection/before closure; *n* = 34	no medication; N = 33
Kolasiński 2026 [12]	A randomized, triple-blinded, placebo-controlled clinical trial	Poland	October 2023 and October 2025	patients aged 18–75 years old with ASA I-II who underwent elective abdominoplasty	60	TXA 10 mg/kg IV over 10 min; completed ≥10 min before incision; *n* = 30	Placebo; N = 30
Manfellotto 2025 [24]	Retrospective cohort	Italy	May 2022 and May 2024	patients who underwent abdominoplasty with umbilical transposition	221 reported; 211 arm-assigned	TXA 1 g IV preoperatively; *n* = 181	no medication; N = 30
Sahinovic 2025 [5]	Retrospective cohort	Germany	January 2020 and June 2025	patients who underwent abdominoplasty	58	TXA 1 g IV at incision, followed by source-reported “1-1-1” for 48 h; oral continuation after early discharge; *n* = 24	no medication; N = 34
Zucal 2025 [26]	Retrospective cohort	Switzerland	June 2020 and September 2023.	patients who underwent abdominoplasty	60	TXA 1 g topical in 20 mL of saline via two drains after closure; drains clamped for 20 min; *n* = 30	no medication; N = 30
Ciccarelli 2026 [22]	Retrospective pilot	Italy	January 2023 and December 2024	patients who underwent abdominoplasty	200	TXA 1 g IV at induction + 500 mg of local infiltration; 2 g IV on POD1 (two doses) + 1 g IV on POD2; *n* = 100	no medication; N = 100
Gorji 2026 [23]	Retrospective cohort	Germany	January 2015 to October 2025	patients who underwent abdominoplasty	97	TXA 1 g IV at surgery start; *n* = 49	no medication; N = 48

**Note.** The seven source cohorts reported 763 participants. Treatment-arm assignment was available for 753. Manfellotto 2025 reported 221 participants overall but provided arm-level counts for only 181 TXA-treated and 30 untreated participants (n = 211), leaving 10 without extractable TXA-arm classification. No allocation was imputed; outcome-specific denominators were used. Clarification by the original study authors would be required to resolve the remaining 10 participants. **Abbreviations:** IV, intravenous; N, number of patients; TXA, tranexamic acid.

**Table 2:** Baseline characteristics of the included studies.

Study ID	Arm	Age, Years	Male Sex, n (%)	BMI, kg/m^2^ (Mean ± SD)	Hypertension, n (%)	Diabetes, n (%)	Smoking, n (%)
Zaussinger 2024 [25]	Tranexamic acid	37.5 (25–63)	7 (20.6%)	25.2 ± 3.9	NA	NA	NA
	No medication	38 (23–68)	3 (9.1%)	26 ± 2.5	NA	NA	NA
Kolasiński 2026 [12]	Tranexamic acid	43.5 ± 9	NA	25.9 ± 3.9	NA	NA	NA
	Placebo	42.9 ± 5	NA	24 ± 3.8	NA	NA	NA
Manfellotto 2025 [24]	All patients	47.5 (27–60) Median (IQR)	74 (33.5%)	NA	NA	NA	69 (31.2%)
Sahinovic 2025 [5]	Tranexamic acid	45 ± 10.6	5 (20.8%)	26.4 ± 4.3	NA	3 (12.5%)	4 (16.7%)
	No medication	47.9 ± 12.6	5 (14.7%)	28.2 ± 4.8	NA	7 (20.5%)	7 (20.5%)
Zucal 2025 [26]	Tranexamic acid	55 ± 9	1 (3.3%)	26.2 ± 4.5	NA	NA	NA
	No medication	54 ± 11	1 (3.3%)	24.6 ± 4.5	NA	NA	NA
Ciccarelli 2026 [22]	Tranexamic acid	42	NA	NA	NA	NA	NA
	No medication	43	NA	NA	NA	NA	NA
Gorji 2026 [23]	Tranexamic acid	41.6 ± 13.5	10 (20.4%)	27.3 ± 3.8	6 (12.2%)	7 (14.3%)	7 (14.3%)
	No medication	42.8 ± 14.1	10 (20.8%)	28.1 ± 4.9	9 (18.8%)	5 (10.4%)	9 (18.8%)

**Abbreviations:** BMI, body mass index; NA, not available; SD, standard deviation.

## Data Availability

All data are provided in the manuscript and its Supplementary Material.

## Supplementary Materials

The following supporting information can be downloaded at https://www.mdpi.com/article/10.3390/jcm15176886/s1, Supplementary Table S1. Search Strategy. Supplementary Table S2. Quality assessment of the included observational studies using NOS. Supplementary Table S3. Study-level TXA regimens and seroma-related co-interventions. Supplementary Table S4. Quality-based sensitivity analysis excluding the poor-quality study. Supplementary Figure S1. Risk-of-bias assessment for the randomized controlled trial using the RoB 2 tool. Supplementary Figure S2. Leave-one-out sensitivity analysis of total drainage volume. Supplementary Figure S3. Doi plot of total drainage volume. Supplementary Figure S4. Leave-one-out sensitivity analysis of drainage time. Supplementary Figure S5. Doi plot of drainage time. Supplementary Figure S6. Leave-one-out sensitivity analysis of absolute hemoglobin drop. Supplementary Figure S7. Doi plot of absolute hemoglobin drop. Supplementary Figure S8. Leave-one-out sensitivity analysis of seroma. Supplementary Figure S9. Doi plot of seroma. Supplementary Figure S10. Leave-one-out sensitivity analysis of hematoma. Supplementary Figure S11. Doi plot of hematoma. Supplementary Figure S12. Leave-one-out sensitivity analysis of wound-healing disorder. Supplementary Figure S13. Doi plot of wound-healing disorder. PRISMA Checklist. Completed PRISMA 2020 checklist for this systematic review and meta-analysis.

## Abbreviations

TXA, tranexamic acid; RCT, randomized controlled trial; MD, mean difference; CI, confidence interval; SSI, surgical-site infection; OR, odds ratio; PRISMA, Preferred Reporting Items for Systematic Reviews and Meta-Analyses; PROSPERO, International Prospective Register of Systematic Reviews; MeSH, Medical Subject Headings; SD, standard deviation; RoB 2, Cochrane Risk of Bias 2 tool; NOS, Newcastle-Ottawa Scale; I^2^, inconsistency statistic; τ^2^, between-study variance; LFK, Luis Furuya–Kanamori index; NICE, National Institute for Health and Care Excellence.
