# Supplementary material for: Efficacy and Safety of Tranexamic Acid in Abdominoplasty: A Systematic Review and Meta-Analysis
Source: J Clin Med. 2026 Sep 5;15(17):6886. doi: 10.3390/jcm15176886 (PMC13565830; doi:10.3390/jcm15176886)
Supplement: Supplementary file 1 [file jcm-15-06886-s001.zip › jcm-4478233-supplementary.pdf]

Supplementary Materials

Supplementary Table S1. Search Strategy.

Supplementary Table S2. Quality assessment of the included observational studies using NOS.

Supplementary Figure S1. Risk-of-bias assessment for the randomized controlled trial using the RoB 2 tool.

Supplementary Figure S2. Leave-one-out sensitivity analysis of total drainage volume.

Supplementary Figure S3. Doi plot of total drainage volume.

Supplementary Figure S4. Leave-one-out sensitivity analysis of drainage time.

Supplementary Figure S5. Doi plot of drainage time.

Supplementary Figure S6. Leave-one-out sensitivity analysis of absolute hemoglobin drop.

Supplementary Figure S7. Doi plot of absolute hemoglobin drop.

Supplementary Figure S8. Leave-one-out sensitivity analysis of seroma.

Supplementary Figure S9. Doi plot of seroma.

Supplementary Figure S10. Leave-one-out sensitivity analysis of hematoma.

Supplementary Figure S11. Doi plot of hematoma.

Supplementary Figure S12. Leave-one-out sensitivity analysis of wound-healing disorder.

Supplementary Figure S13. Doi plot of wound-healing disorder.

Supplementary Table S3. Study-level TXA regimens and seroma-related co-interventions.

Supplementary Table S4. Quality-based sensitivity analysis excluding the poor-quality study.

PRISMA Checklist

Supplementary Table S1. Search Strategy

Database	Search Strategy	N	Limitations	Search date
**PubMed**	(abdominoplasty OR Panniculectomy OR Abdominoplasties OR Panniculectomies OR "Abdominoplasty"[Mesh])<br><br>AND<br><br>(tranexamic acid OR Transamin OR Amchafibrin OR Ugurol OR Cyklokapron OR Anvitoff OR Spotof OR Exacyl OR "Tranexamic Acid"[Mesh])	22	No limitations applied	16 May 2026
**Scopus**	(abdominoplasty OR Panniculectomy OR Abdominoplasties OR Panniculectomies)<br><br>AND<br><br>(tranexamic acid OR Transamin OR Amchafibrin OR Ugurol OR Cyklokapron OR Anvitoff OR Spotof OR Exacyl)	28	Title, abstract, keywords	16 May 2026
**Web of Science**	(abdominoplasty OR Panniculectomy OR Abdominoplasties OR Panniculectomies)<br><br>AND<br><br>(tranexamic acid OR Transamin OR Amchafibrin OR Ugurol OR Cyklokapron OR Anvitoff OR Spotof OR Exacyl)	24	No limitations applied	16 May 2026
**Cochrane Library**	(abdominoplasty OR Panniculectomy OR Abdominoplasties OR Panniculectomies)<br><br>AND<br><br>(tranexamic acid OR Transamin OR Amchafibrin OR Ugurol OR Cyklokapron OR Anvitoff OR Spotof OR Exacyl)	5	No limitations applied	16 May 2026
**Number of studies before removal of duplicates**		79		
**Number of studies after removal of duplicates**		43		

Supplementary Table S2. Quality assessment of the included observational studies using NOS

Study ID	Selection				Comparability	Outcome			Quality Score
	Representativeness of the exposed cohort	Selection of the non-exposed cohort	Ascertainment of exposure	Demonstration that outcome of interest was not present at start of study	Comparability of cohorts on the basis of the design or analysis	Assessment of outcome	Was follow-up long enough for outcomes to occur	Adequacy of follow up of cohorts	
Zaussinger 2024	*	*	*	*	**	*	*	*	Good
Manfellotto 2025	*			*		*	*	*	Poor
Sahinovic 2025	*	*	*	*	**	*	*	*	Good
Zucal 2025	*	*	*	*	**	*		*	Good
Ciccarelli 2026	*	*	*	*	**	*		*	Good
Gorji 2026	*	*	*	*	**	*	*	*	Good

Supplementary Table S3. Study-level TXA regimens and seroma-related co-interventions

Study	TXA regimen	Surgical approach and related measures	Drain management	Other seroma-related measures
Zaussinger 2024	Bodyweight-adapted 1–2 g IV (mean 1.5 g), after resection and before closure; surgeon discretion for diffuse bleeding.	No concomitant liposuction; thin adipose layer beneath Scarpa fascia preserved.	Two 15-Fr Blake suction drains; remove at <30 mL/24 h, earliest POD2.	No PTS; abdominal binder/compression reported; fibrin sealant NR.
Kolasiński 2025	Single 10 mg/kg IV dose in 100 mL saline over 10 min, completed ≥10 min before incision; no further doses.	Standardized procedure; no tumescent solution or liposuction; meticulous electrocautery.	Two closed-suction drains; removal threshold NR.	Quilting/PTS, Scarpa preservation, compression, and fibrin sealant NR.
Manfellotto 2025	1 g IV preoperatively.	Scarpa fascia preservation; tumescent infiltration; manual/scalpel dissection; avoidance of electrocautery described.	Two CH10 Redon drains; remove at ≤20 mL/24 h.	No quilting sutures; three tension-relieving vectors; binder for 3 weeks and compression stockings.
Sahinovic 2025	1 g IV at incision, then source-reported "1-1-1" for 48 h; oral continuation after early discharge.	Conventional or fleur-de-lis surgery; liposuction and rectus repair as indicated; rectus repair imbalanced (62.5% TXA vs 26.5% control).	Redon drains in all cases; remove at <30 mL/24 h.	Standardized compression; PTS varied by surgeon; Scarpa preservation and fibrin sealant NR.
Zucal 2025	1 g topical TXA in 20 mL saline through two drains after closure; drains clamped for 20 min.	Electrocautery; mixed abdominoplasty indications/types; almost no liposuction.	Two 10-Ch drains; remove at approximately ≤30 mL/24 h; discharge after removal.	No quilting sutures; Scarpa preservation, compression, and fibrin sealant NR.
Ciccarelli 2026	1 g IV at induction + 500 mg local infiltration; 2 g IV on POD1 in two doses + 1 g IV on POD2.	Standardized post-bariatric abdominoplasty with liposuction and rectus plication.	Drain details and removal threshold NR.	Postoperative compression garment; quilting/PTS, Scarpa preservation, and fibrin sealant NR.
Gorji 2026	1 g IV at surgery start.	Procedures performed by 4–5 surgeons over 10 years; detailed liposuction/tumescent/electrocautery technique NR.	Drains used; removal based on output and clinical judgment, with no strict predefined threshold.	Quilting/PTS, Scarpa preservation, compression, and fibrin sealant NR.

Note. IV, intravenous; NR, not reported; POD, postoperative day; PTS, progressive-tension sutures; TXA, tranexamic acid. The Sahinovic source describes a "1-1-1" regimen for 48 hours without further unit clarification; the source wording is retained. Absence of reporting was not interpreted as absence of the co-intervention.

Supplementary Table S4. Quality-based sensitivity analysis excluding the poor-quality study

Analysis	Studies	Participants	MD [95% CI], g/dL	p value	I^2
Primary absolute-hemoglobin-drop meta-analysis	3	508	−0.87 [−1.72, −0.02]	0.0461	95.3%
Excluding Manfellotto 2025 (poor quality)	2	297	−0.60 [−1.81, 0.62]	0.336	98%

Note. CI, confidence interval; MD, mean difference; TXA, tranexamic acid. Manfellotto 2025 was the only poor-quality study and contributed only to the absolute-hemoglobin-drop meta-analysis; therefore, no other pooled outcome required a quality-based exclusion analysis.

Supplementary Figure S1. Risk-of-bias assessment for the randomized controlled trial using the RoB 2 tool.

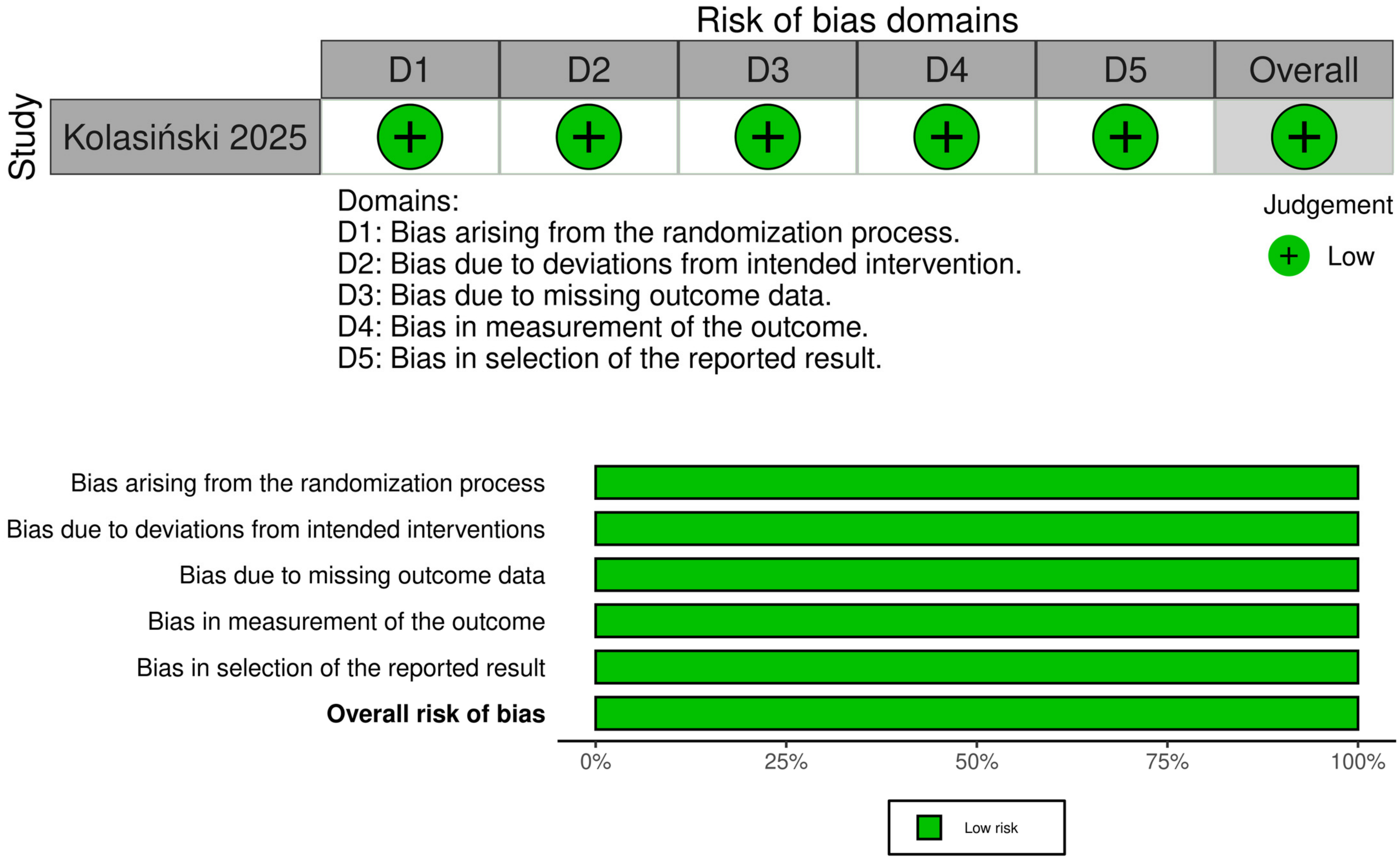

Supplementary Figure S2. Leave-one-out sensitivity analysis of total drainage volume.

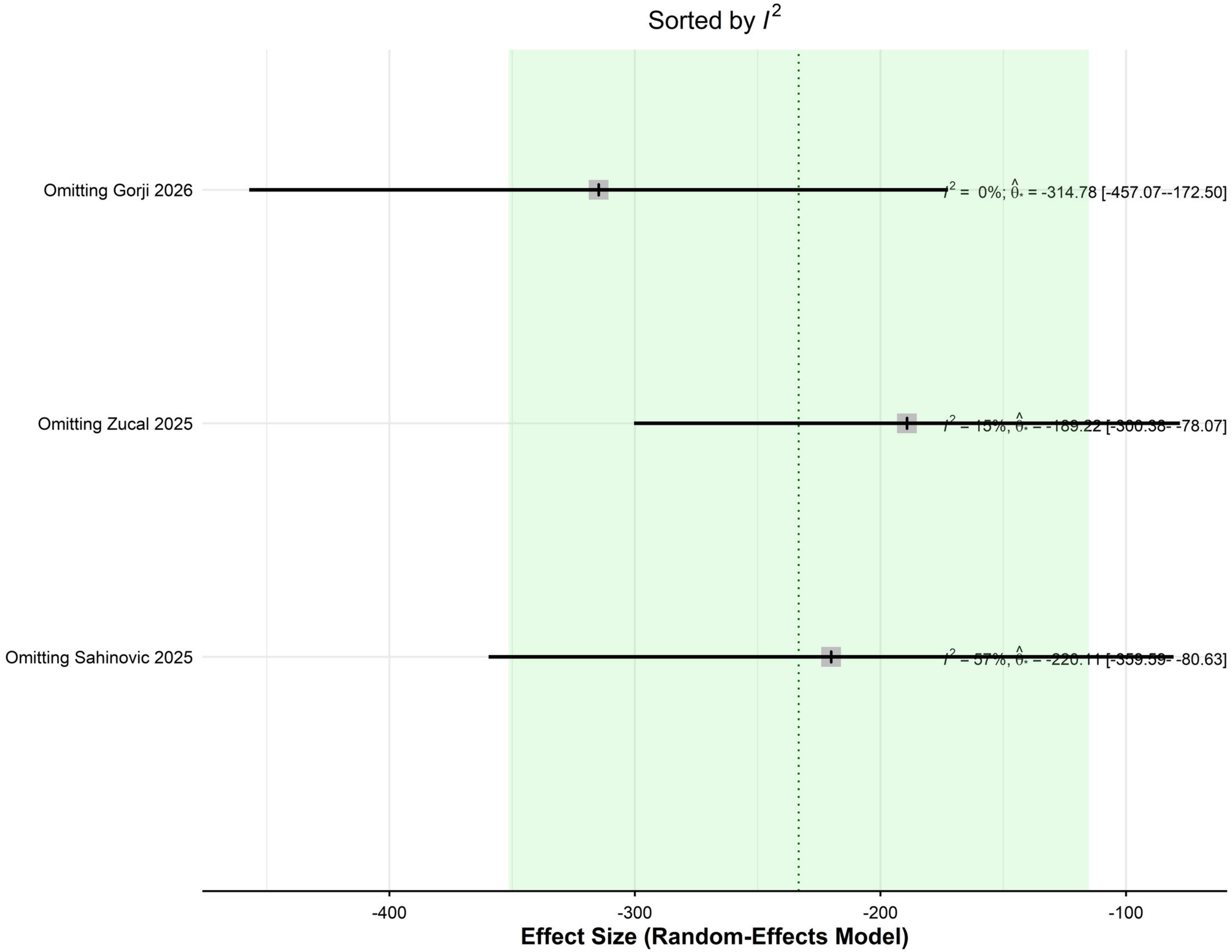

Supplementary Figure S3. Doi plot of total drainage volume (LFK index = -5.45).

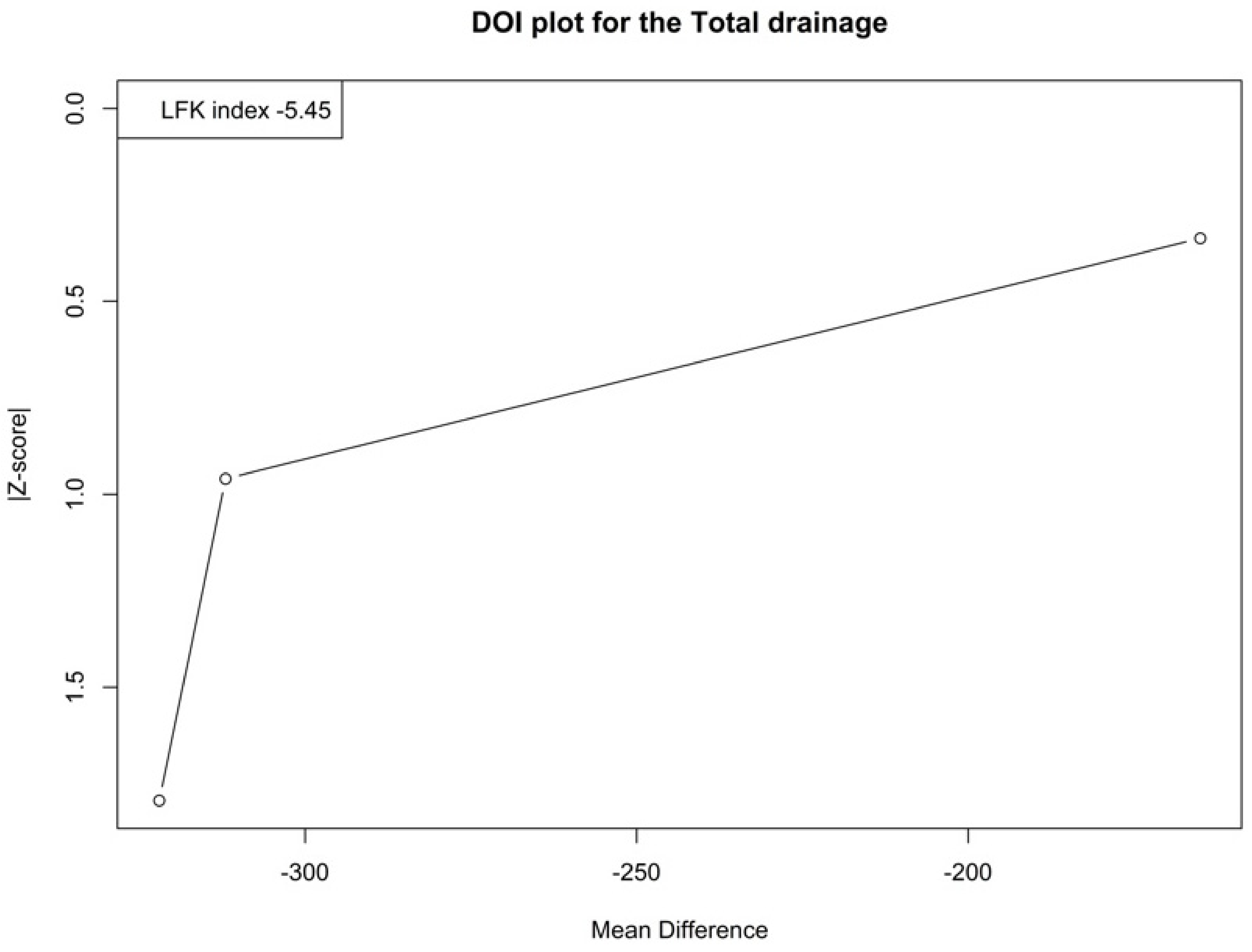

Supplementary Figure S4. Leave-one-out sensitivity analysis of drainage time.

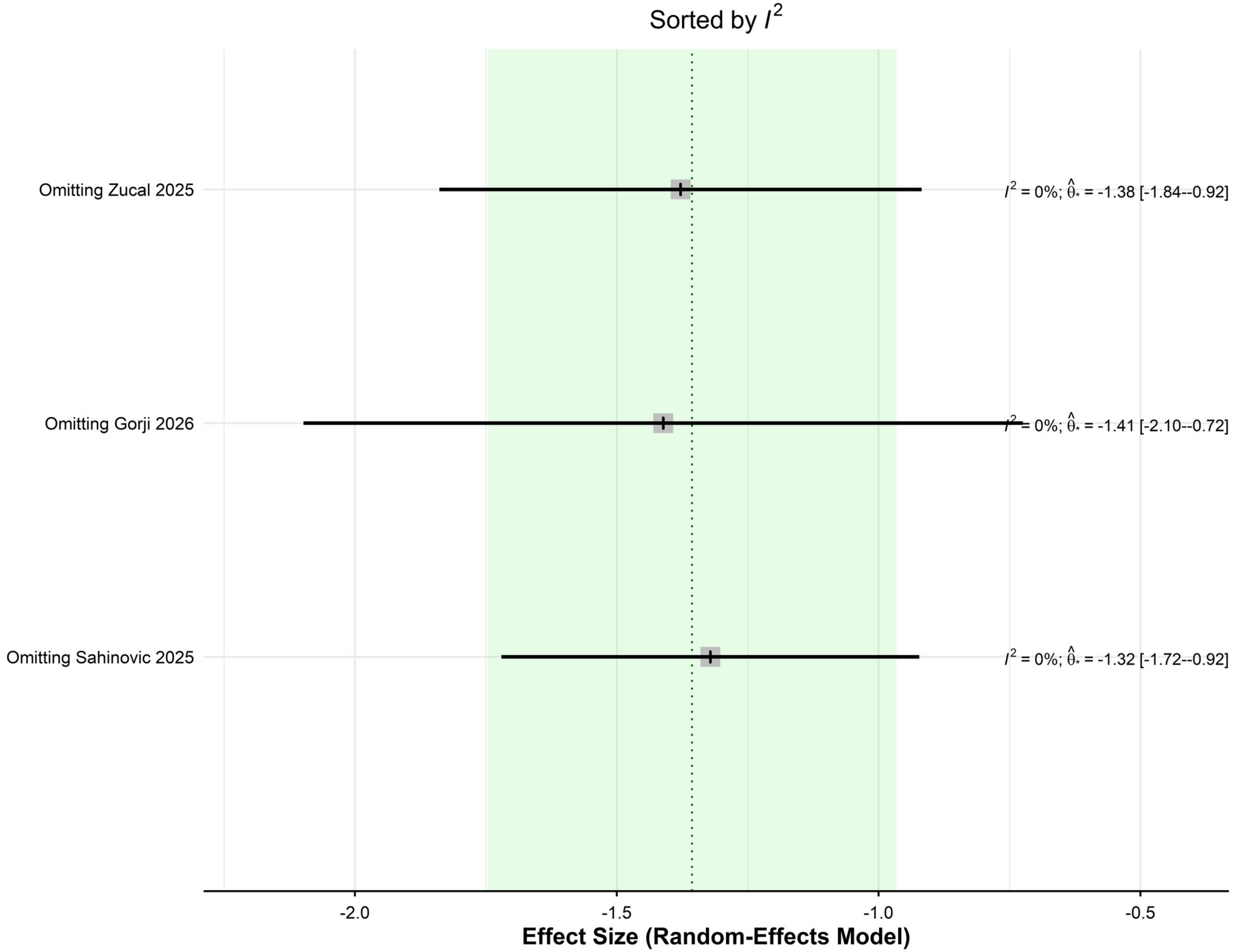

Supplementary Figure S5. Doi plot of drainage time (LFK index = -3.46).

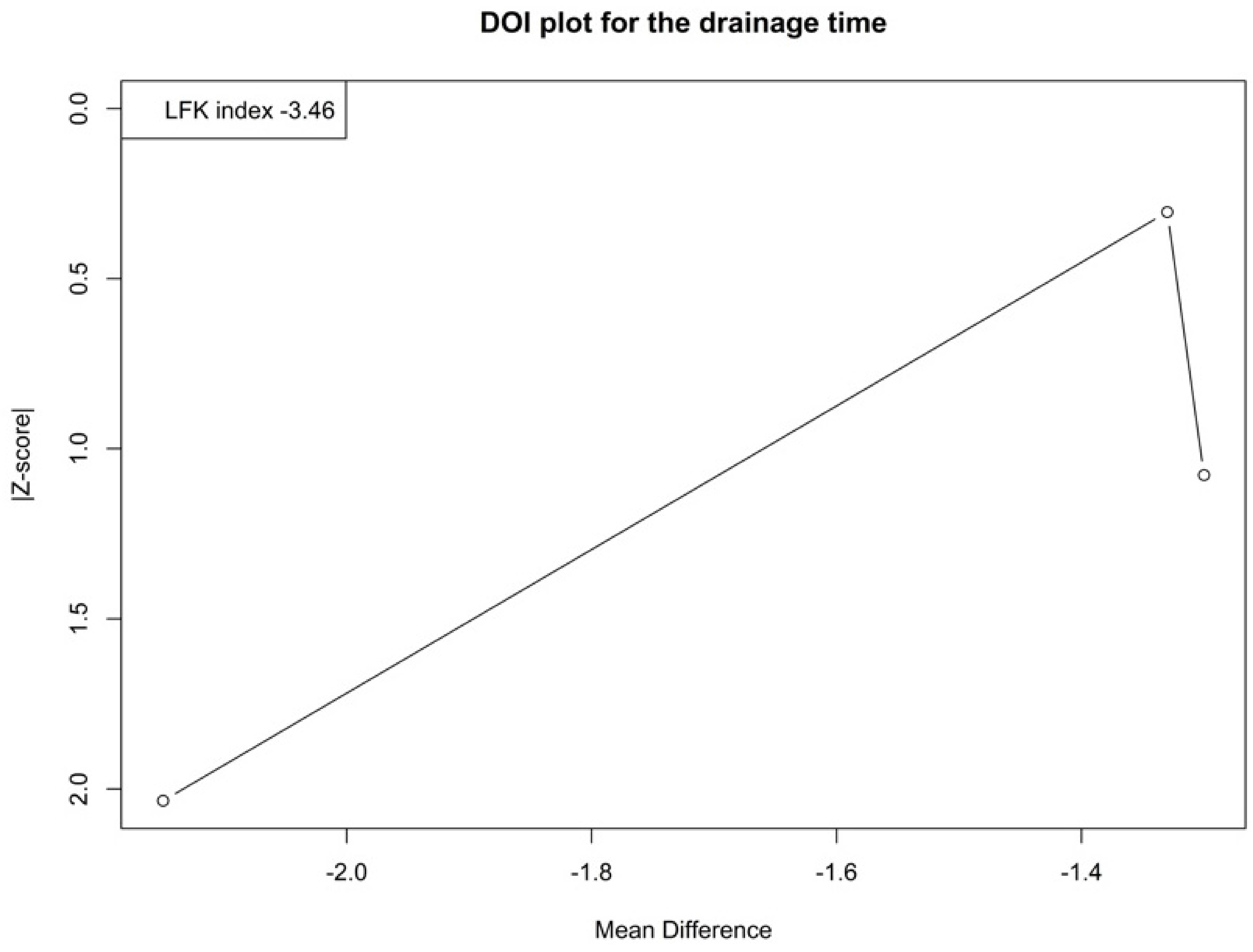

Supplementary Figure S6. Leave-one-out sensitivity analysis of absolute hemoglobin drop. The analysis omitting Manfellotto 2025 also represents the quality-based sensitivity analysis excluding the only poor-quality study and yielded MD = −0.60 g/dL (95% CI −1.81 to 0.62; I^2 = 98%). Manfellotto 2025 contributed to no other pooled outcome.

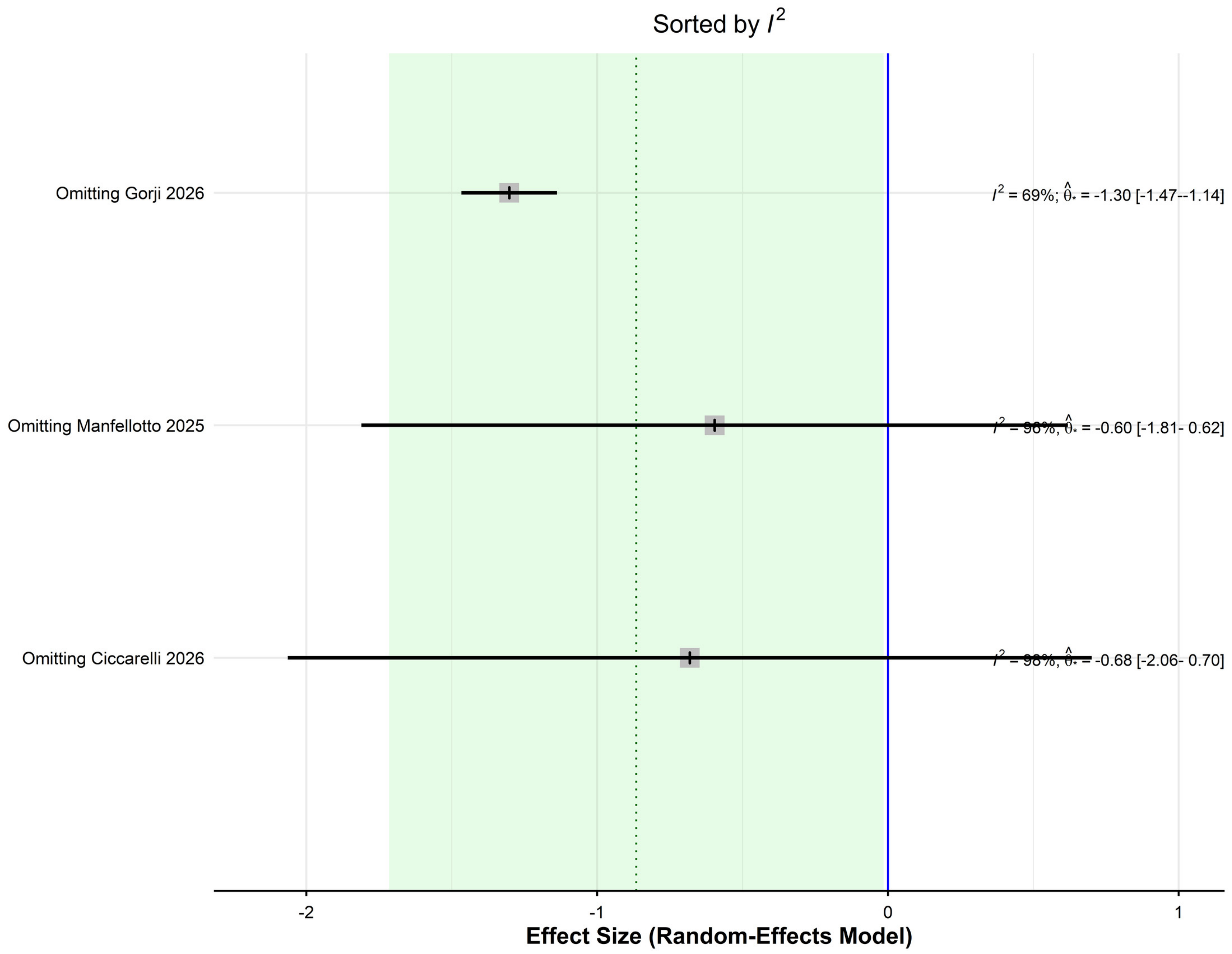

Supplementary Figure S7. Doi plot of absolute hemoglobin drop (LFK index = 4.94).

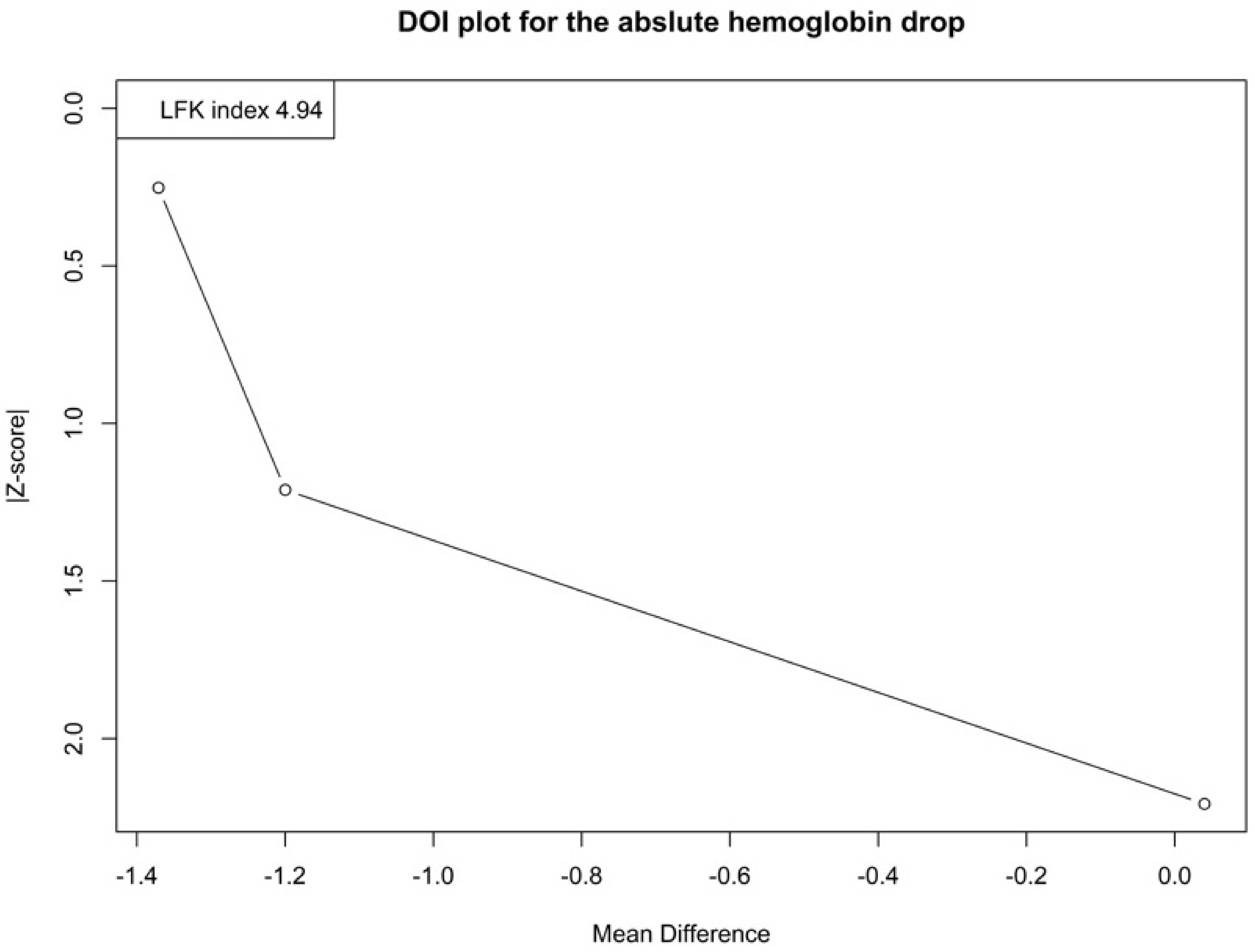

Supplementary Figure S8. Leave-one-out sensitivity analysis of seroma.

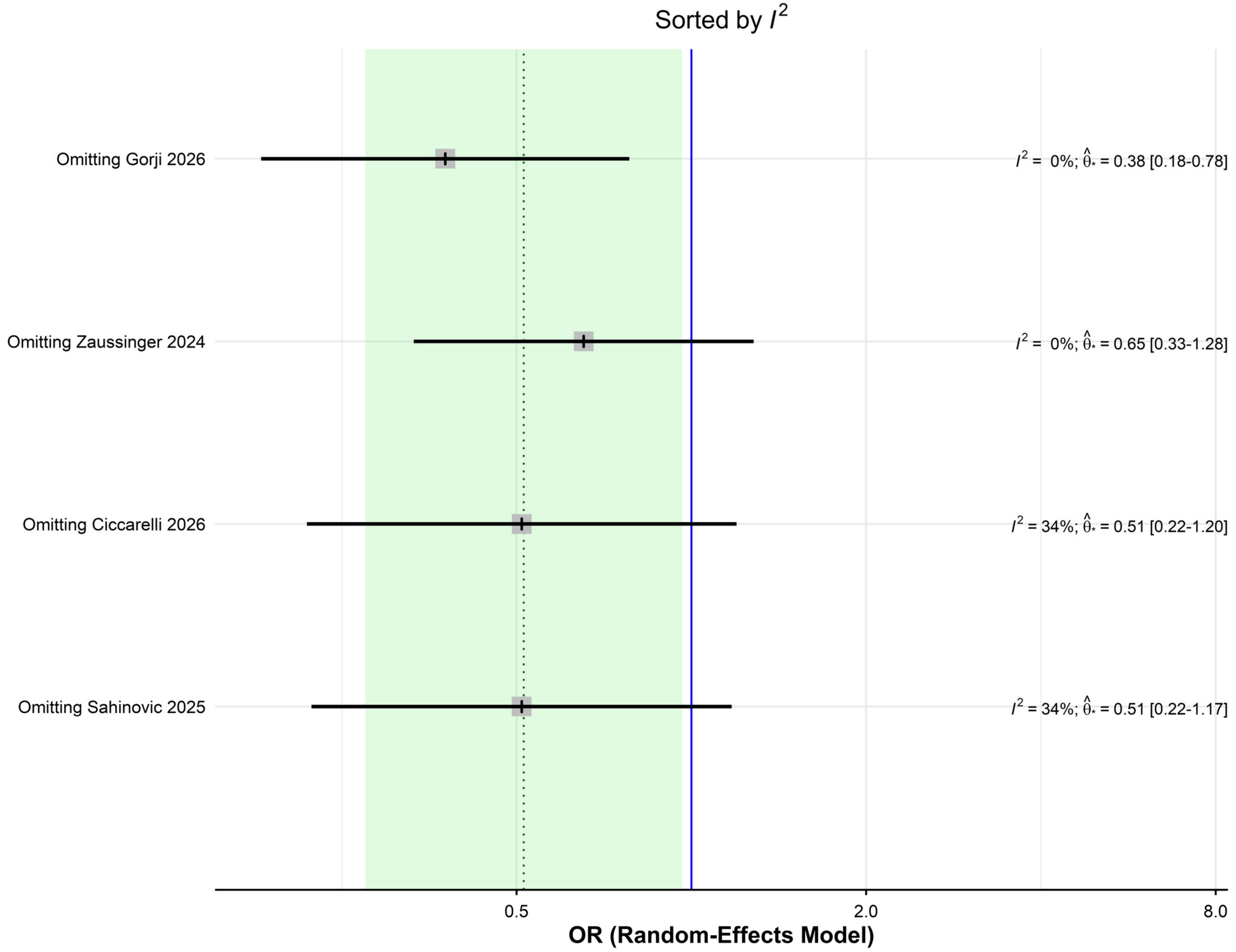

Supplementary Figure S9. Doi plot of seroma (LFK index = -0.36).

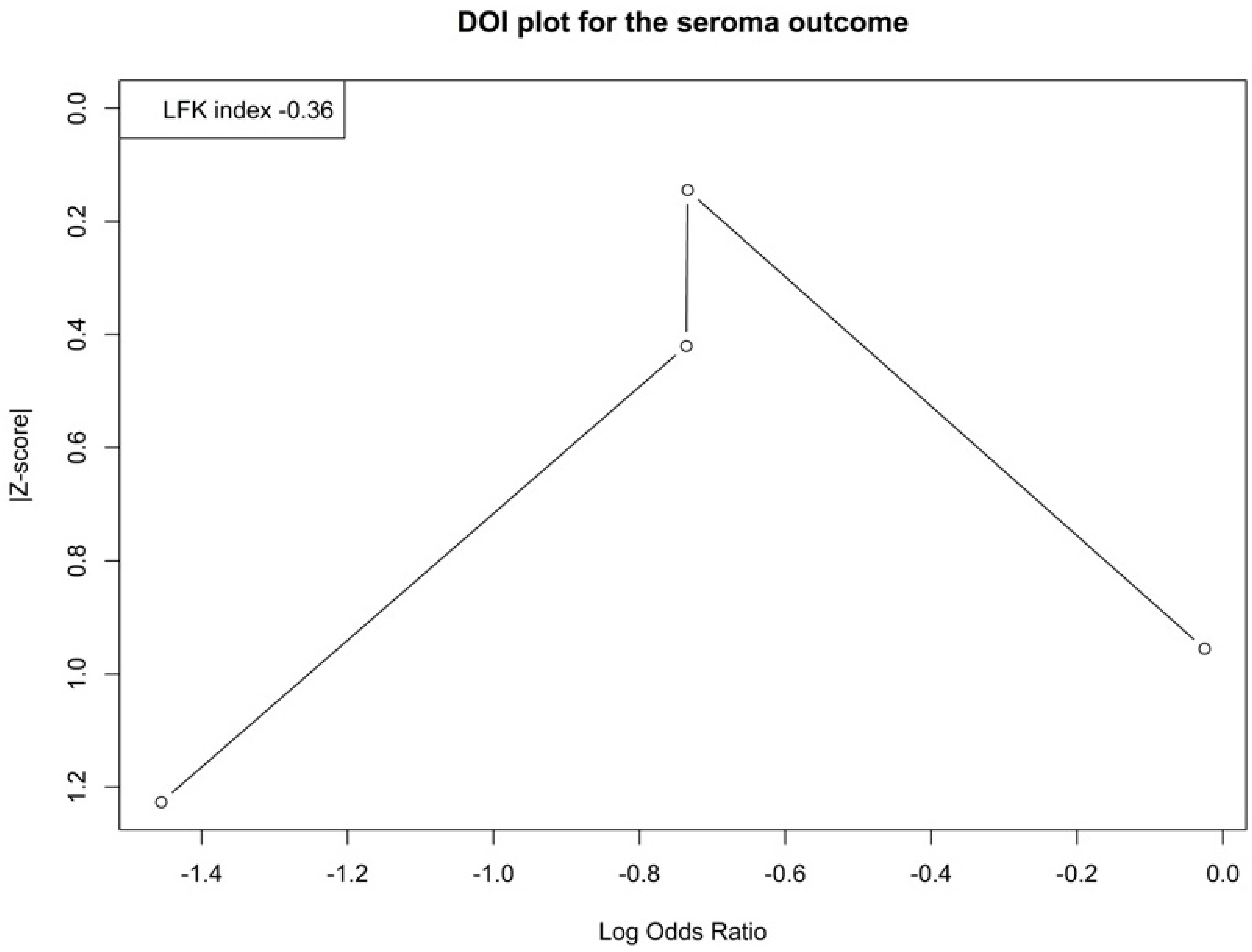

Supplementary Figure S10. Leave-one-out sensitivity analysis of hematoma.

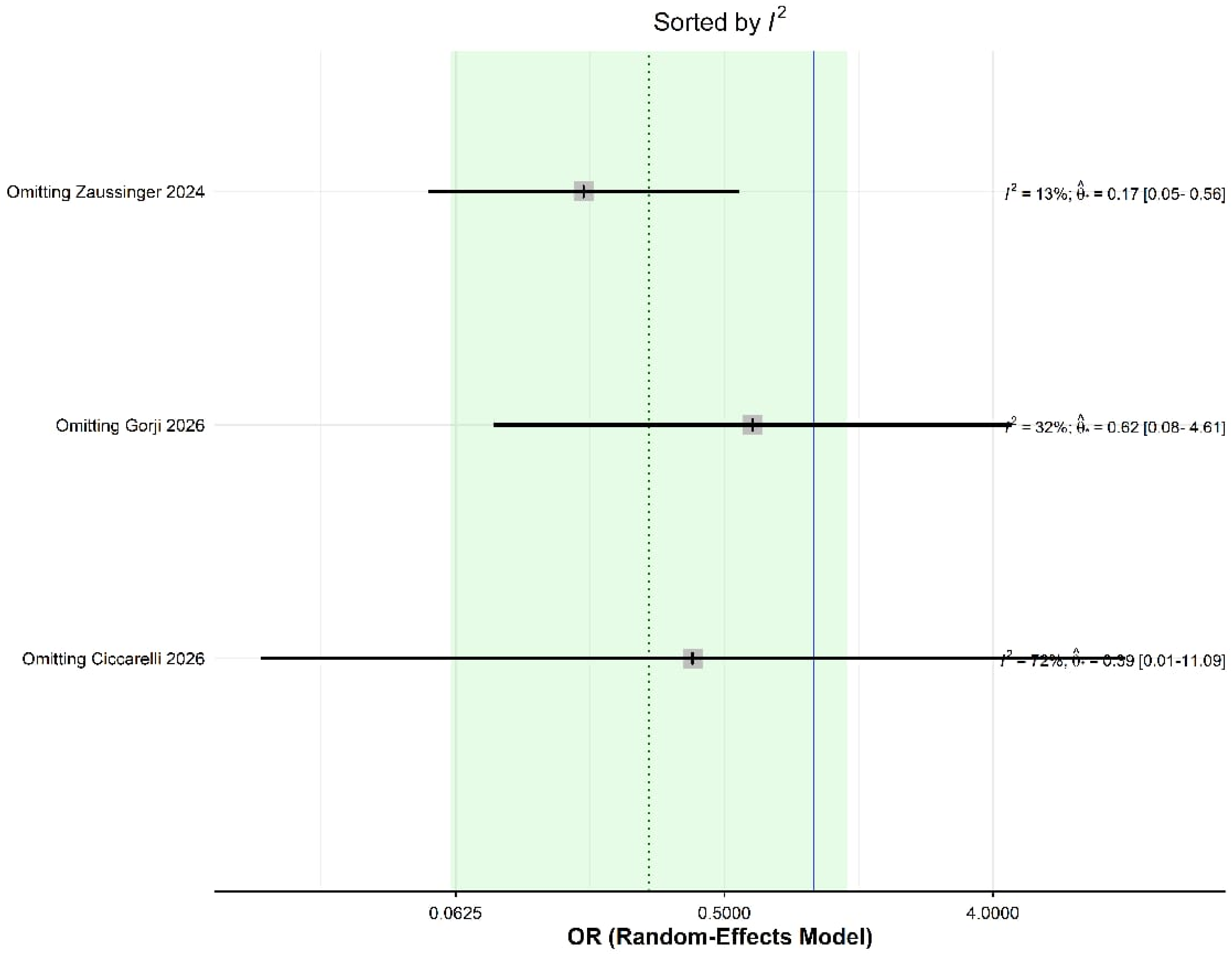

Supplementary Figure S11. Doi plot of hematoma (LFK index = 1.70).

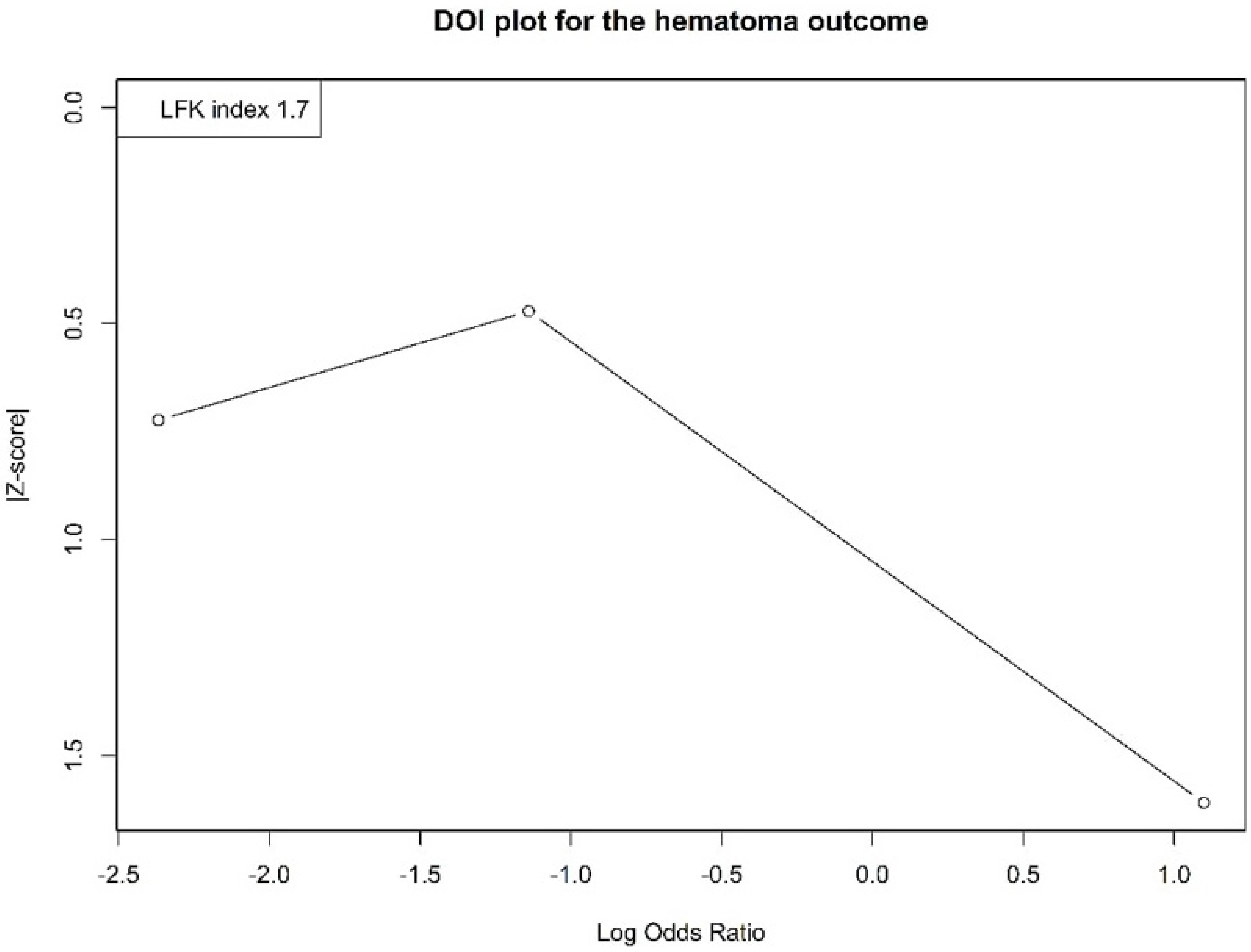

Supplementary Figure S12. Leave-one-out sensitivity analysis of wound-healing disorder.

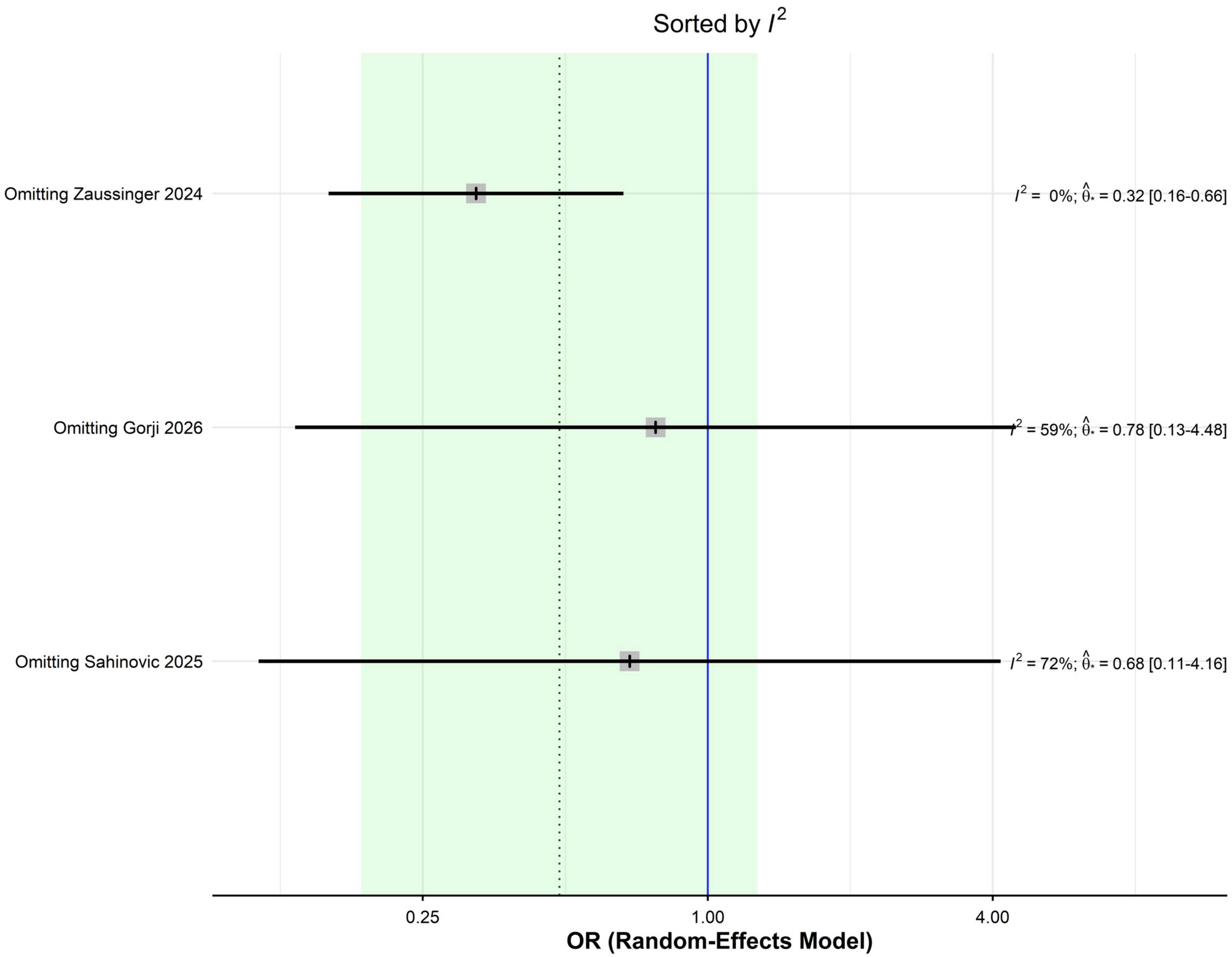

Supplementary Figure S13. Doi plot of wound-healing disorder (LFK index = 3.07).

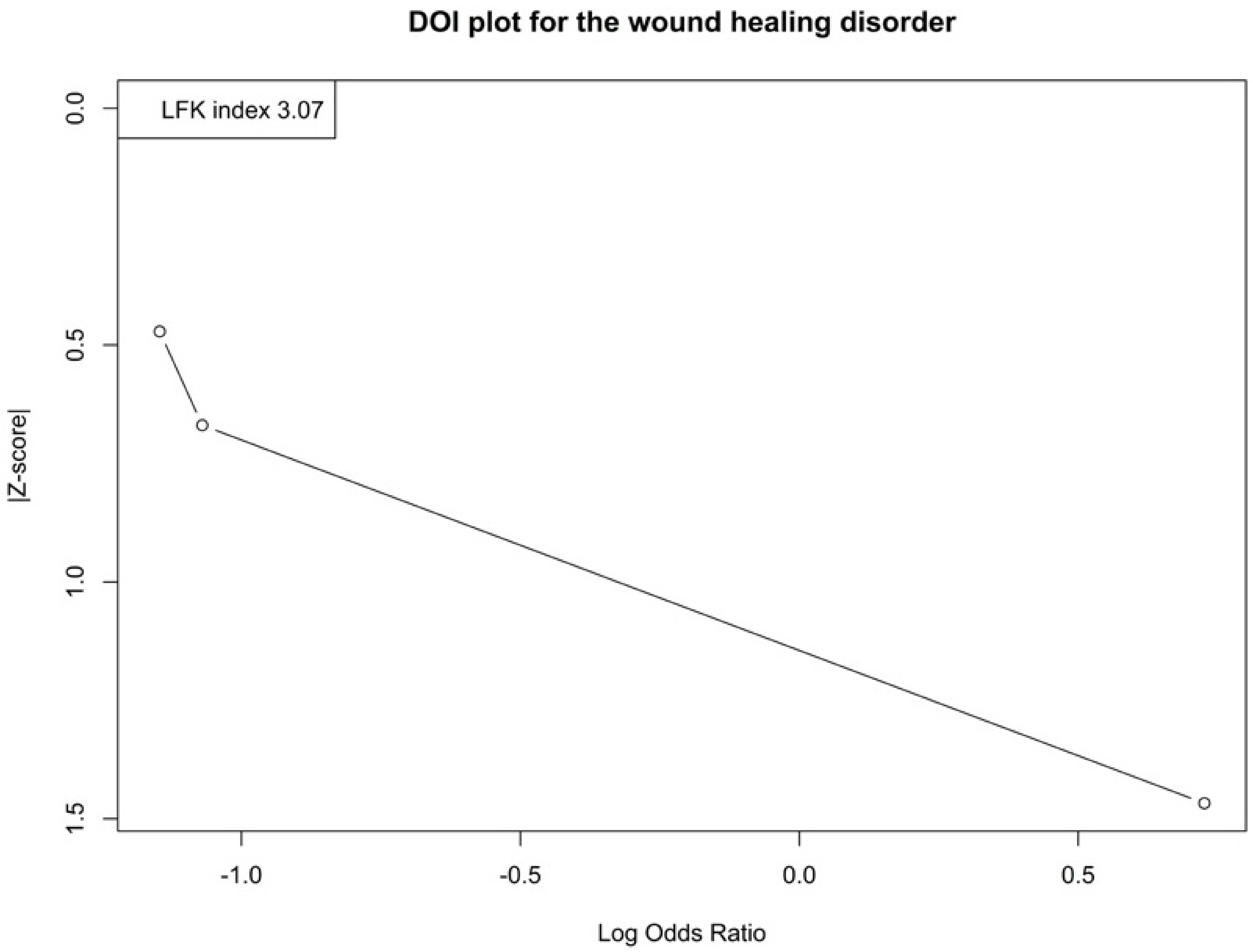

PRISMA Checklist

Section and Topic	Item #	Checklist item	Location where item is reported
**TITLE**			
Title	1	Identify the report as a systematic review.	1
**ABSTRACT**			
Abstract	2	See the PRISMA 2020 for Abstracts checklist.	1–2
**INTRODUCTION**			
Rationale	3	Describe the rationale for the review in the context of existing knowledge.	2–3
Objectives	4	Provide an explicit statement of the objective(s) or question(s) the review addresses.	3
**METHODS**			
Eligibility criteria	5	Specify the inclusion and exclusion criteria for the review and how studies were grouped for the syntheses.	4–5
Information sources	6	Specify all databases, registers, websites, organisations, reference lists and other sources searched or consulted to identify studies. Specify the date when each source was last searched or consulted.	3
Search strategy	7	Present the full search strategies for all databases, registers and websites, including any filters and limits used.	3; Supplementary Table S1
Selection process	8	Specify the methods used to decide whether a study met the inclusion criteria of the review, including how many reviewers screened each record and each report retrieved, whether they worked independently, and if applicable, details of automation tools used in the process.	4–5
Data collection process	9	Specify the methods used to collect data from reports, including how many reviewers collected data from each report, whether they worked independently, any processes for obtaining or confirming data from study investigators, and if applicable, details of automation tools used in the process.	4–5
Data items	10a	List and define all outcomes for which data were sought. Specify whether all results that were compatible with each outcome domain in each study were sought (e.g. for all measures, time points, analyses), and if not, the methods used to decide which results to collect.	4–5
	10b	List and define all other variables for which data were sought (e.g. participant and intervention characteristics, funding sources). Describe any assumptions made about any missing or unclear information.	4–5; Supplementary Table S3
Study risk of bias assessment	11	Specify the methods used to assess risk of bias in the included studies, including details of the tool(s) used, how many reviewers assessed each study and whether they worked independently, and if applicable, details of automation tools used in the process.	5; Supplementary Figure S1 and Supplementary Table S2
Effect measures	12	Specify for each outcome the effect measure(s) (e.g. risk ratio, mean difference) used in the synthesis or presentation of results.	5
Synthesis methods	13a	Describe the processes used to decide which studies were eligible for each synthesis (e.g. tabulating the study intervention characteristics and comparing against the planned groups for each synthesis (item #5)).	4–5
	13b	Describe any methods required to prepare the data for presentation or synthesis, such as handling of missing summary statistics, or data conversions.	4–5
	13c	Describe any methods used to tabulate or visually display results of individual studies and syntheses.	8–12; Figures 2–5
	13d	Describe any methods used to synthesize results and provide a rationale for the choice(s). If meta-analysis was performed, describe the model(s), method(s) to identify the presence and extent of statistical heterogeneity, and software package(s) used.	5
	13e	Describe any methods used to explore possible causes of heterogeneity among study results (e.g. subgroup analysis, meta-regression).	Not performed
	13f	Describe any sensitivity analyses conducted to assess robustness of the synthesized results.	5, 8–11; Supplementary Figures 2, 4, 6, 8, 10, and 12; Supplementary Table S4
Reporting bias assessment	14	Describe any methods used to assess risk of bias due to missing results in a synthesis (arising from reporting biases).	5
Certainty assessment	15	Describe any methods used to assess certainty (or confidence) in the body of evidence for an outcome.	Not performed
**RESULTS**			
Study selection	16a	Describe the results of the search and selection process, from the number of records identified in the search to the number of studies included in the review, ideally using a flow diagram.	5–6

Section and Topic	Item #	Checklist item	Location where item is reported
	16b	Cite studies that might appear to meet the inclusion criteria, but which were excluded, and explain why they were excluded.	5–6
Study characteristics	17	Cite each included study and present its characteristics.	6–7; Tables 1–2; Supplementary Table S3
Risk of bias in studies	18	Present assessments of risk of bias for each included study.	7; Supplementary Figure S1 and Supplementary Table S2
Results of individual studies	19	For all outcomes, present, for each study: (a) summary statistics for each group (where appropriate) and (b) an effect estimate and its precision (e.g. confidence/credible interval), ideally using structured tables or plots.	8–12; Figures 2–5
Results of syntheses	20a	For each synthesis, briefly summarise the characteristics and risk of bias among contributing studies.	6–12
	20b	Present results of all statistical syntheses conducted. If meta-analysis was done, present for each the summary estimate and its precision (e.g. confidence/credible interval) and measures of statistical heterogeneity. If comparing groups, describe the direction of the effect.	8–12; Figures 2–5
	20c	Present results of all investigations of possible causes of heterogeneity among study results.	Not performed
	20d	Present results of all sensitivity analyses conducted to assess the robustness of the synthesized results.	8–11; Supplementary Figures 2, 4, 6, 8, 10, and 12; Supplementary Table S4
Reporting biases	21	Present assessments of risk of bias due to missing results (arising from reporting biases) for each synthesis assessed.	8–11 and 15; Supplementary Figures 3, 5, 7, 9, 11, and 13
Certainty of evidence	22	Present assessments of certainty (or confidence) in the body of evidence for each outcome assessed.	Not performed
**DISCUSSION**			
Discussion	23a	Provide a general interpretation of the results in the context of other evidence.	13–15
	23b	Discuss any limitations of the evidence included in the review.	15
	23c	Discuss any limitations of the review processes used.	15
	23d	Discuss implications of the results for practice, policy, and future research.	15
**OTHER INFORMATION**			
Registration and protocol	24a	Provide registration information for the review, including register name and registration number, or state that the review was not registered.	3
	24b	Indicate where the review protocol can be accessed, or state that a protocol was not prepared.	3
	24c	Describe and explain any amendments to information provided at registration or in the protocol.	Not reported
Support	25	Describe sources of financial or non-financial support for the review, and the role of the funders or sponsors in the review.	16
Competing interests	26	Declare any competing interests of review authors.	16
Availability of data, code and other materials	27	Report which of the following are publicly available and where they can be found: template data collection forms; data extracted from included studies; data used for all analyses; analytic code; any other materials used in the review.	16
